# Supplementary material for: The host–guest inclusion driven by host-stabilized charge transfer for construction of sequentially red-shifted mechanochromic system
Source: Nat Commun. 2023 Jul 13;14:4190. doi: 10.1038/s41467-023-39956-7 (PMC10345137; doi:10.1038/s41467-023-39956-7)
Supplement: Supplementary file 2 — Supplementary Information [file 41467_2023_39956_MOESM2_ESM.pdf]

SUPPLEMENTARY INFORMATION

The Host-guest Inclusion Driven by Host-stabilized Charge Transfer for Construction of Sequentially Red-shifted Mechanochromic System

Dongdong Sun, [1] *Yong Wu,* [1] *Xie Han* [1, 2] *and Simin Liu** [1, 2]

[1] School of Chemistry and Chemical Engineering, Wuhan University of Science and Technology, Wuhan 430081, China.

[2] The State Key Laboratory of Refractories and Metallurgy, Institute of Advanced Materials and Nanotechnology, Wuhan University of Science and Technology, Wuhan 430081, China.

* Corresponding author, E-mail: *liusimin@wust.edu.cn*

Table of Contents

Synthesis and characterization 3

Fluorescence spectra of **G1** in different solvents 15

MCL behavior of **G1-G3** 16

Fluorescence lifetime decay profiles 17

ITC data for **G1** with CB[8] 19

Host-guest recognition of CB[8] and **G2**/**G3** 19

MCL behavior of CB[8]·$\mathbf{G}_2$ 22

Crystal structure analysis 23

Solid state absorption spectra of **G1** and CB[8]·$\mathbf{G1}_2$ 24

Host-guest recognition of CB[8] and **Py** 25

Host-guest recognition and MCL behavior of CB[8]·**Py**·**G1** 26

Crystal data 33

Theoretical calculations 36

Supplementary References 47

Synthesis and characterization

4-Bromotriphenylamine(98%), iodomethane(99.5%), glycoluril(97%) ammonium hexafluorophosphate(95%) were purchased from Bide Pharmatech Ltd. 4-Pyridinylboronic acid(96%), paraformaldehyde(95%), phosphorus oxychloride(99%), tetrakis(triphenylphosphine)palladium(0) (Pd≥8.9%), potassium carbonate(99%), 1-pyrenemethylamine hydrochloride(97%) and Amberlite® IRA-400(Cl) resin were purchased from Anhui Senrise Technology Co., Ltd (Energy Chemical). N,N-Dimethylformamide (≥99.5%), dichloromethane (≥99.5%), tetrahydrofuran (≥99.5%), trichloromethane (≥99.5%), acetonitrile (≥99.5%), methanol (≥99.5%), concentrated hydrochloric acid (content: 36.0% ~ 38.0%) and dimethyl sulfoxide (≥99.5%) were purchased from Sinopharm Chemical Reagent Co., Ltd. Deuterium solvents were purchased from Aldrich. All reagents were purchased from commercial suppliers and used without further purification.

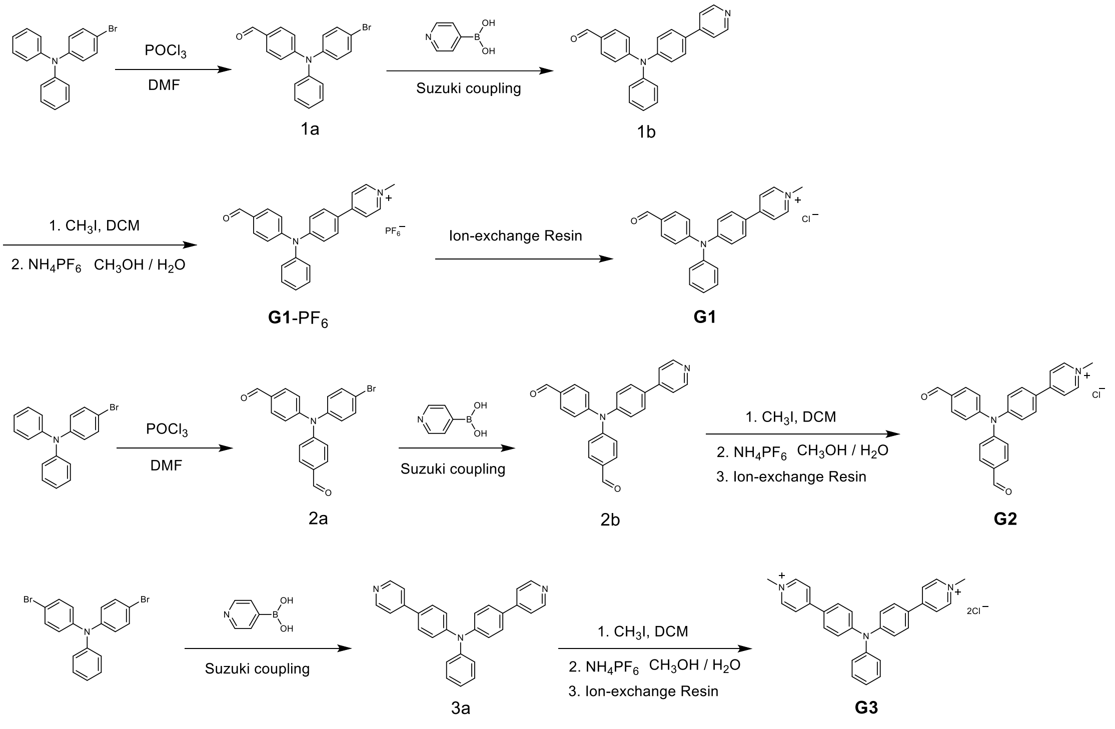

Supplementary Figure 1. Synthetic routes for **G1**-**G3**.

Synthesis of **1a** were adapted and modified from previously reported procedures.[1]
4-Bromotriphenylamine (1.0 g, 3.08 mmol) was dissolved in 15 mL DMF, phosphorus oxychloride (0.35 mL, 3.70 mmol) was then injected to the solution. The mixture was stirred at 383 K overnight. Then the mixture was poured into 150 mL H_2O, and the solid was collected and purified by means of column chromatography (petroleum ether/ethyl acetate 20:1) to give compound **1a** as a green solid (0.77 g, 71%).
1a: ^{1}H NMR (600 MHz, DMSO-d_6) δ = 9.79 (s, 1H), 7.78 – 7.67 (m, 2H), 7.62 – 7.53 (m, 2H), 7.47 – 7.38 (m, 2H), 7.27 – 7.22 (m, 1H), 7.21 – 7.17 (m, 2H), 7.13 – 7.09 (m, 2H), 6.97 – 6.92 (m, 2H).

*Synthesis of **1b**:*

Potassium carbonate (0.63 g, 4.54 mmol) and tetrakis(triphenylphosphine)palladium(0) (0.26 g, 0.23 mmol) were added to a stirred solution of **1a** (0.80 g, 2.27 mmol) and 4-pyridinylboronic acid (0.56 g, 4.54 mmol) in degassed THF (80 mL). The mixture was heated under reflux for 24 h under the protection of N_2 gas and then concentrated. The mixture was then evaporated and purified by means of column chromatography (petroleum ether/ethyl acetate 4:1) to give compound **1b** as a yellow solid (0.63 g, 79%).

1b: ^{1}H NMR (600 MHz, DMSO-d_6) δ = 9.81 (s, 1H), 8.68 – 8.58 (m, 2H), 7.88 – 7.81 (m, 2H), 7.81 – 7.74 (m, 2H), 7.74 – 7.68 (m, 2H), 7.45 (t, *J* = 7.9 Hz, 2H), 7.27 (dd, *J* = 8.1, 6.4 Hz, 3H), 7.24 – 7.18 (m, 2H), 7.07 – 6.97 (m, 2H).

^{13}C NMR (150 MHz, DMSO-d_6) δ = 191.17, 150.67, 132.45, 131.94, 131.88, 131.74, 130.59, 129.21, 129.13, 128.72, 127.03, 126.18, 126.12, 121.23, 120.04.

HRMS (m/z): m/z calcd for $[M + H]^+$ $[C_{24}H_{19}N_2O]^+$: 351.1492; found: 351.1521, calcd for $[M + Na]^+$ 373.1311; found: 373.1329.

*Synthesis of **G1**-PF$_6$:*

1b (0.50 g, 1.43 mmol) was dissolved in dry DCM (10 mL) and CH_3I (1 mL) was injected. The mixture was stirred at room temperature for 12 h. Then acquired yellow solid and the reaction mixture was filtered, washed with DCM and dried in vacuum to give yellow solid. Then the yellow solid was dissolved in MeOH:H_2O (1:1, 20 mL), added excess NH_4PF_6 to the solution and the filter cake was filtered, the obtained solid was washed with H_2O for three times in order to wash off excess NH_4PF_6, dried in vacuum at 60°C to give green solid (0.45 g, 86%).

G1-PF$_6$**:** ^{1}H NMR (600 MHz, DMSO-d_6) δ = 9.87 (s, 1H), 8.93 (d, *J* = 6.2 Hz, 2H), 8.43 (d, *J* = 6.3 Hz, 2H), 8.08 (d, *J* = 8.3 Hz, 2H), 7.84 (d, *J* = 8.2 Hz, 2H), 7.48 (t, *J* = 7.7 Hz, 2H), 7.32 (t, *J* = 7.4 Hz, 1H), 7.25 (t, *J* = 8.5 Hz, 4H), 7.15 (d, *J* = 8.2 Hz, 2H), 4.29 (s, 3H).

^{13}C NMR (150 MHz, DMSO-d_6) δ = 196.28, 158.37, 156.76, 154.76, 150.61, 150.29, 136.56, 135.80, 135.55, 134.93, 132.94, 132.19, 131.51, 129.08, 128.24, 126.84, 52.04.

*Synthesis of **G1**-Cl:*

The yellow solid **G1**-Cl was given in yield of 90% after thorough counter anion exchange in water using Amberlite® IRA-400(Cl) resin (by simply shaking the suspension of the **G1**-PF$_6$ and resin in water overnight) and lyophilization.

G1-Cl**:** ^{1}H NMR (600 MHz, D_2O) δ = 9.72 (s, 1H), 8.68 (d, *J* = 6.4 Hz, 2H), 8.21 (d, *J* = 6.3 Hz, 2H), 7.89 (d, *J* = 8.4 Hz, 2H), 7.83 – 7.72 (m, 2H), 7.46 (t, *J* = 7.5 Hz, 2H), 7.40 – 7.28 (m, 4H), 7.23 (d, *J* = 7.9 Hz, 2H), 7.18 (d, *J* = 8.3 Hz, 2H), 4.34 (s, 3H).

^{13}C NMR (150 MHz, D_2O) δ = 195.10, 156.42, 154.53, 151.60, 147.41, 147.31, 133.98, 132.58, 132.18, 131.75, 130.68, 129.27, 128.76, 126.45, 125.93, 123.68, 49.64.

HRMS (ESI; m/z): [**G1**]$^+$ calcd. for $[C_{25}H_{21}N_2O]^+$, 365.1648; found, 365.1648.

Synthesis of **2a**/**2b** was adapted and modified from previously reported procedures.[2] 4-Bromotriphenylamine (1.0 g, 3.08 mmol) was dissolved in 15 mL DMF, phosphorus oxychloride (2.5 mL, 26.46 mmol) was then injected to the solution. The mixture was stirred at 383 K overnight. Then the mixture was poured into 150 mL H_2O, and the solid was collected and purified by means of column chromatography (petroleum ether/ethyl acetate 12:1) to give compound **2a** as a green solid (0.88 g, 75%).

2a: ^{1}H NMR (600 MHz, DMSO-*d*$_6$) δ = 9.89 (s, 2H), 7.85 (d, *J* = 8.5 Hz, 4H), 7.63 (d, *J* = 8.7 Hz, 2H), 7.19 (d, *J* = 8.5 Hz, 4H), 7.16 – 7.13 (m, 2H).

Potassium carbonate (1.16 g, 8.41 mmol) and Tetrakis(triphenylphosphine)palladium(0) (0.24 g, 0.21 mmol) were added to a stirred solution of **2a** (0.8 g, 2.10 mmol) and 4-pyridinylboronic acid (0.52 g, 4.21 mmol) in degassed THF (80 mL). The mixture was heated under reflux for 24 h under the protection of N_2 gas and then concentrated. The mixture was then evaporated and purified by means of column chromatography to give compound **2b** as a yellow solid (0.65 g, 82%).

2b: ^{1}H NMR (600 MHz, DMSO-*d*$_6$) δ = 9.90 (s, 2H), 8.65 – 8.62 (m, 2H), 7.88 (tt, *J* = 9.2, 2.3 Hz, 6H), 7.74 – 7.71 (m, 2H), 7.31 (d, *J* = 8.6 Hz, 2H), 7.26 – 7.22 (m, 4H).

The synthesis procedures of **G2**-PF_6 and **G2**-Cl were similar to that of **G1**.

G2-Cl**:** ^{1}H NMR (600 MHz, D_2O) δ = 9.81 (s, 2H), 8.80 – 8.64 (m, 2H), 8.33 – 8.19 (m, 2H), 8.04 – 7.80 (m, 6H), 7.47 – 7.36 (m, 2H), 7.36 – 7.19 (m, 4H), 4.36 (s, 3H).

^{13}C NMR (150 MHz, DMSO-d$_6$) δ = 191.89, 153.48, 151.22, 149.28, 145.93, 132.47, 131.88, 130.37, 129.74, 126.06, 124.50, 123.83, 47.35.

HRMS (ESI; m/z): [**G2**]$^+$ calcd. for $[C_{25}H_{21}N_2O_2]^+$, 393.1598; found, 393.1598.

Synthesis of **3a**/**G3** was adapted and modified from previously reported procedures.[3] Potassium carbonate (1.03 g, 7.44 mmol) and tetrakis(triphenylphosphine)palladium(0) (0.21 g, 0.12 mmol) were added to a stirred solution of 4-Bromo-N-(4-bromophenyl)-N-phenylaniline (0.50 g, 1.24 mmol) and 4-pyridinylboronic acid (0.31 g, 2.48 mmol) in degassed THF (60 mL). The mixture was heated under reflux for 24 h under the protection of N_2 gas and then concentrated. The mixture was then evaporated and purified by means of column chromatography to give compound **3a** as a light yellow solid (0.32 g, 65%).

3a: ^{1}H NMR (600 MHz, DMSO-*d*$_6$) δ = 8.62 – 8.57 (m, 4H), 7.82 – 7.76 (m, 4H), 7.70 – 7.66 (m, 4H), 7.40 (t, *J* = 7.7 Hz, 2H), 7.22 – 7.11 (m, 7H).

3a (0.40 g, mmol) was dissolved in dry DCM (15 mL) and CH_3I (0.6 mL) was injected. The mixture was stirred at room temperature for 24 h. Then acquired red solid and the reaction mixture was filtered, washed with DCM and dried in vacuum to give red solid. Then the solid was dissolved in MeOH:H_2O (1:1, 30 mL), added excess NH_4PF_6 to the solution and the filter cake was filtered, the obtained solid was washed with H_2O for three times in order to wash off excess NH_4PF_6, dried in vacuum at 60°C to give yellow

solid. Compound **G3** was obtained (0.36 g, 72%) after thorough counter anion exchange in water using Amberlite® IRA-400(Cl) resin (by simply shaking the suspension of the yellow solid and resin in water for 20 h) and lyophilization.

G3: ^{1}H NMR (600 MHz, D_2O) δ = 8.67 (dt, *J* = 7.4, 1.8 Hz, 4H), 8.23 (dt, *J* = 7.0, 1.8 Hz, 4H), 7.93 (dt, *J* = 8.7, 1.7 Hz, 4H), 7.50 (ddd, *J* = 8.5, 5.3, 1.7 Hz, 2H), 7.35 (dt, *J* = 8.8, 1.8 Hz, 5H), 7.31 (dt, *J* = 7.3, 1.5 Hz, 2H), 4.33 (s, 6H).

Synthesis of CB[8] was adapted from previously reported procedures.[4]

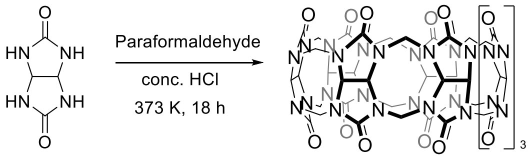

Supplementary Figure 2. Synthetic route for CB[8].

Glycoluril (100 g, 0.7 mol) and paraformaldehyde (42.2 g, 1.4 mol) were added to a two-necked flask, and then conc. HCl (75 mL) was added. The mixture was heated at 373 K for 18 h. The mixture was poured into CH_3OH:H_2O (6:1, 500 mL), the obtained white solid was then washed with water for 5 times (200 mL×5). The resulted white solid was dissolved in 200 mL conc. HCl, and 600 mL H_2O was then added into the solution, the crude product of CB[8] precipitated out of the solution. CB[8] was obtained (7.0 g, 6%) by recrystallization the crude product in 9 M HCl for twice.

CB[8]: ^{1}H NMR (600 MHz, D_2O) δ = 5.77 (d, *J* = 15.7 Hz, 16H), 5.54 (s, 16H), 4.24 (d, *J* = 15.4 Hz, 16H).

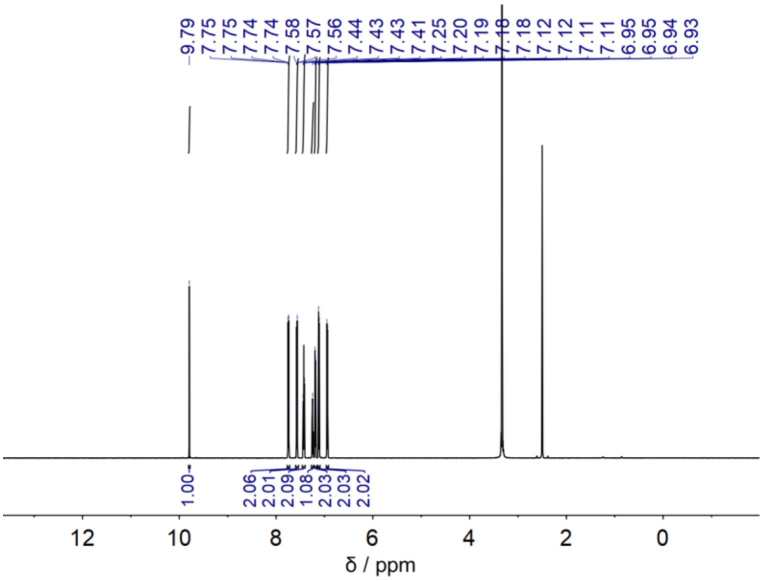

Supplementary Figure 3. ^{1}H NMR spectrum of **1a** (600 MHz, 298 K, DMSO-d_6).

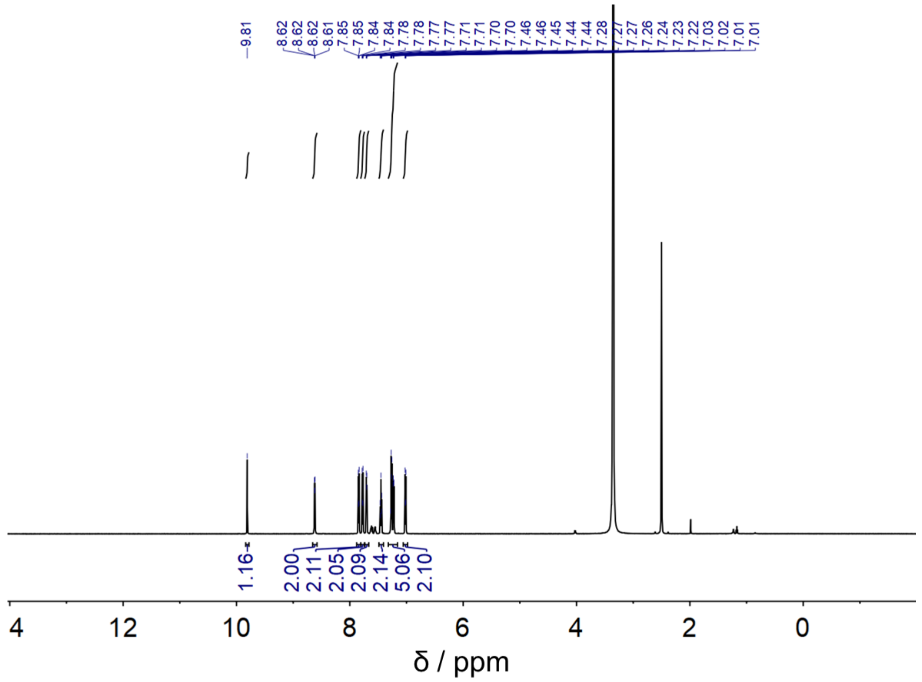

Supplementary Figure 4. ^{1}H NMR spectrum of **1b** (600 MHz, 298 K, DMSO-d_6).

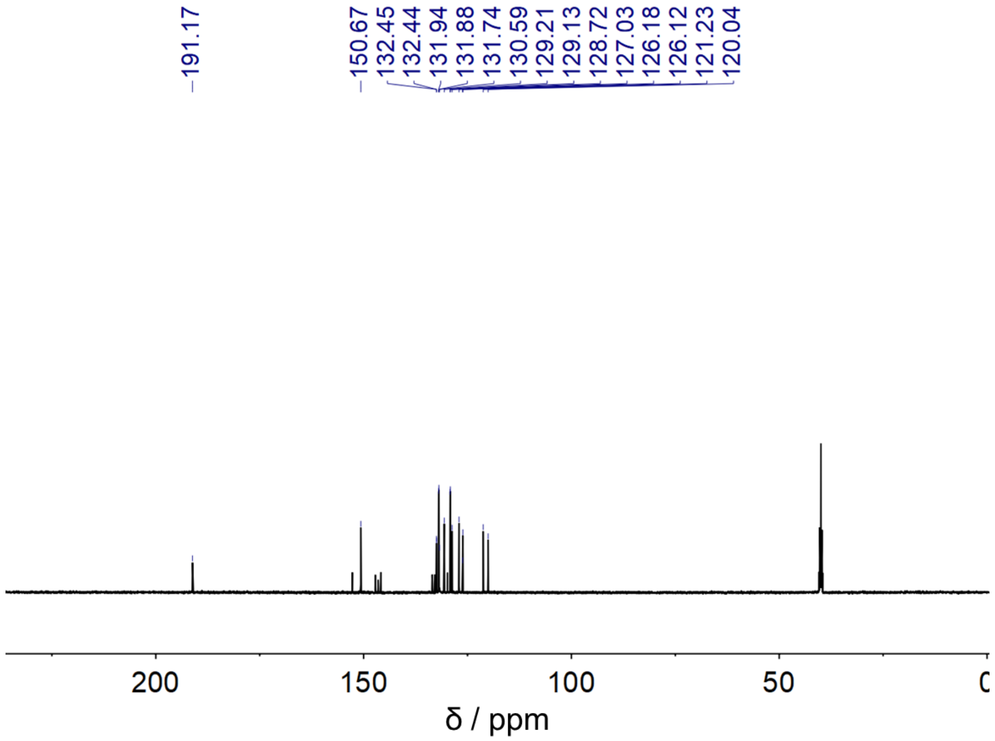

Supplementary Figure 5. ^{13}C NMR spectrum of **1b** (150 MHz, 298 K, DMSO-d_6).

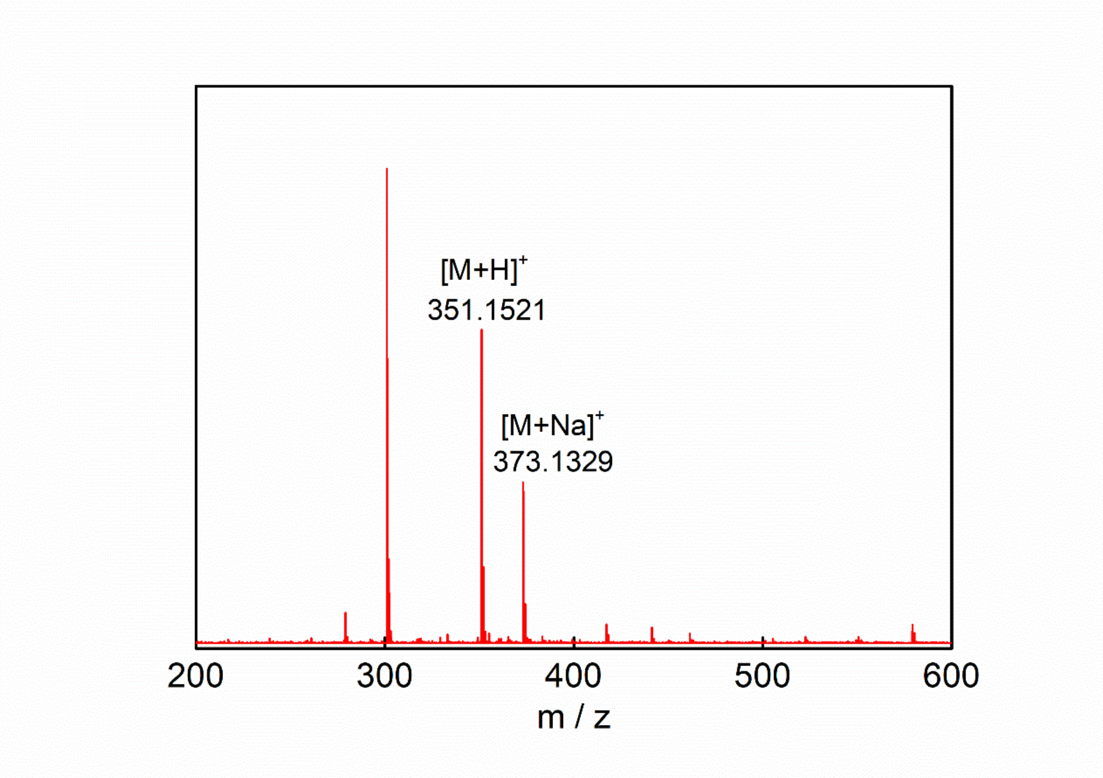

Supplementary Figure 6. HRMS spectrum of **1b**.

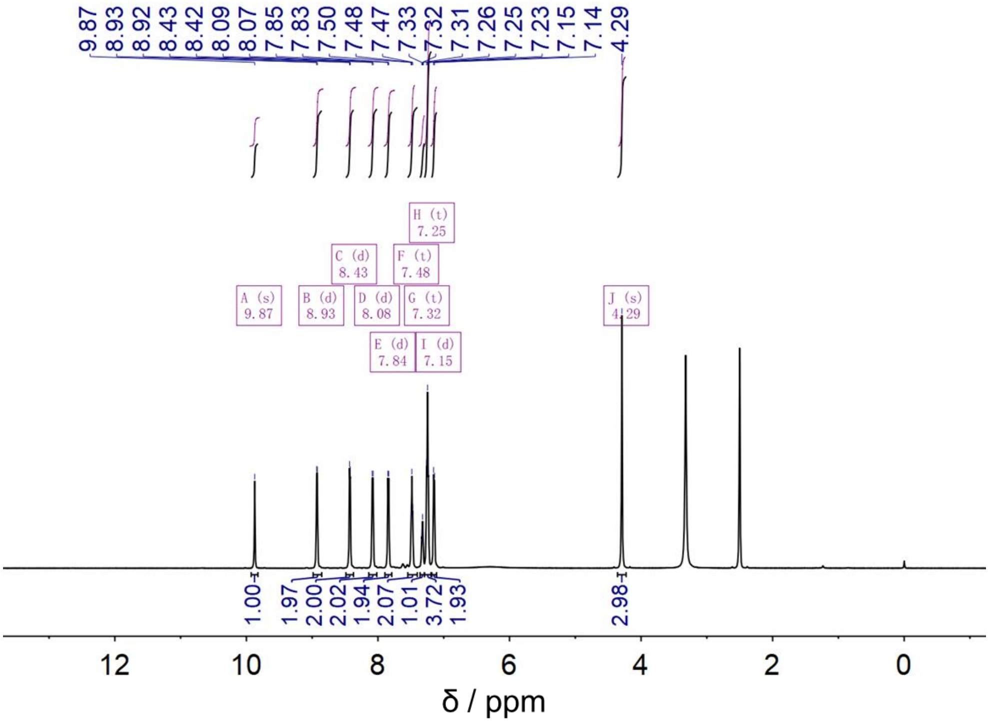

Supplementary Figure 7. ^{1}H NMR spectrum of **G1**-PF_6 (600 MHz, 298 K, DMSO-d_6).

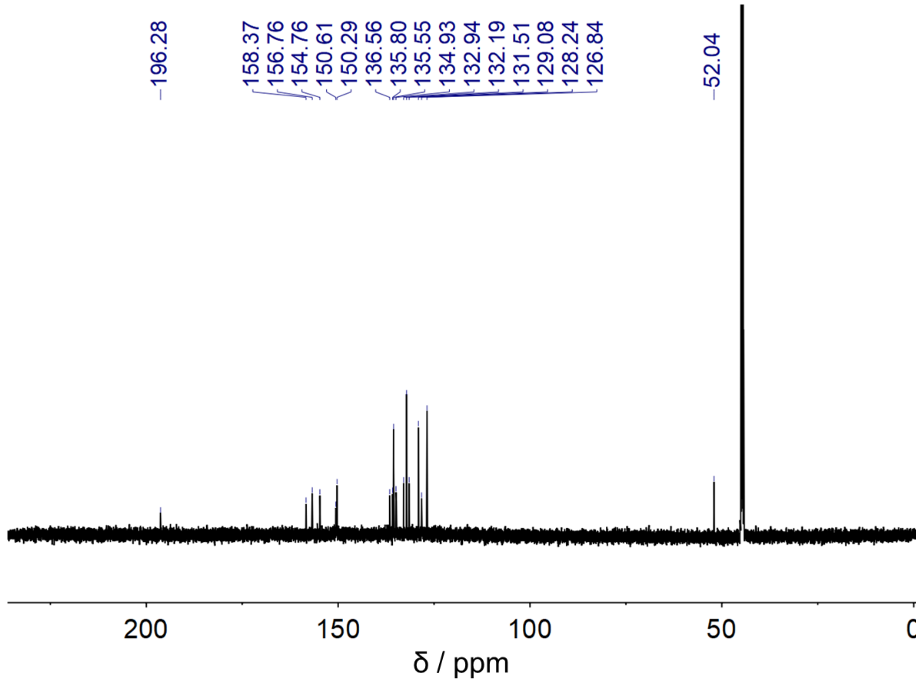

Supplementary Figure 8. ^{13}C NMR spectrum of **G1**-PF_6 (150 MHz, 298 K, DMSO-d_6).

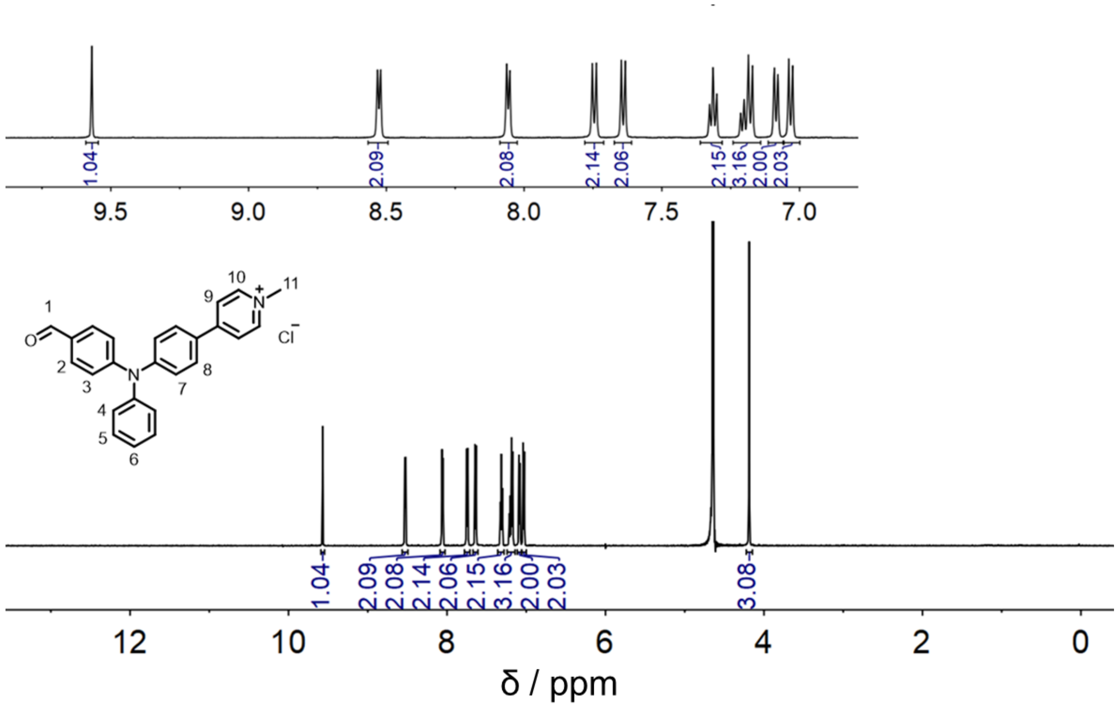

Supplementary Figure 9. ^{1}H NMR spectrum of **G1** (600 MHz, 298 K, D_2O).

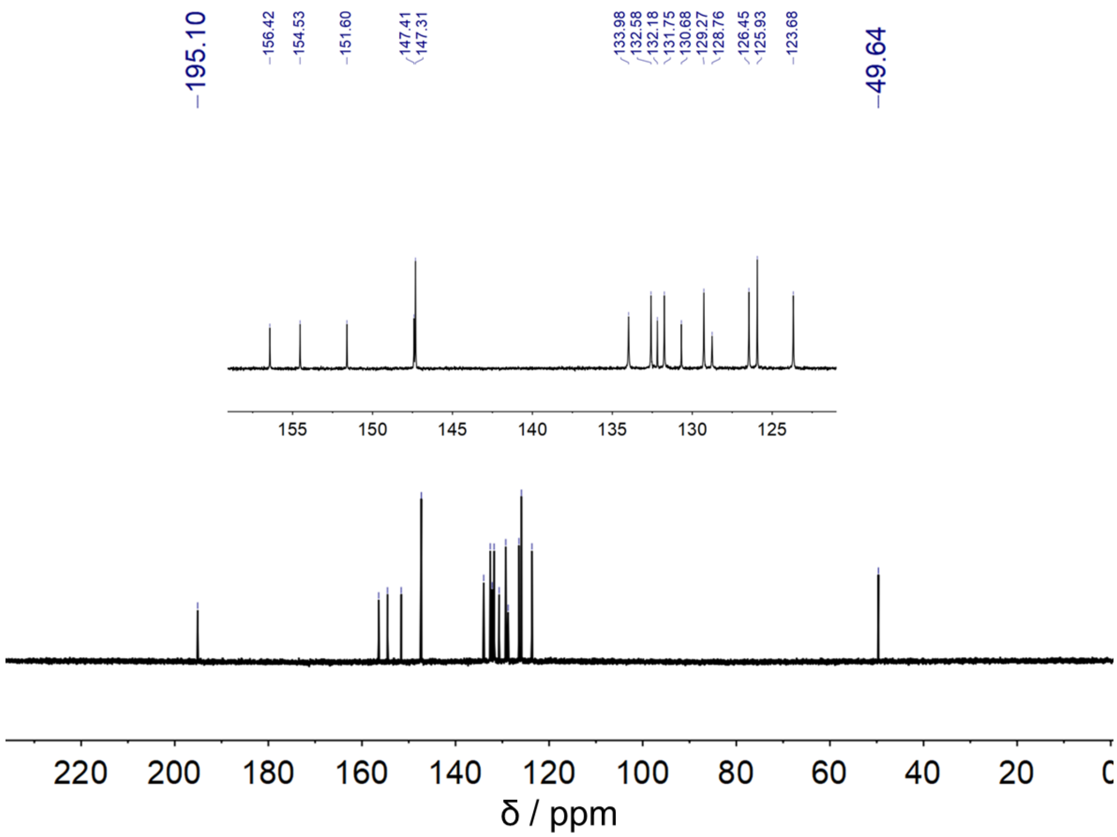

Supplementary Figure 10. ^{13}C NMR spectrum of **G1** (150 MHz, 298 K, D_2O).

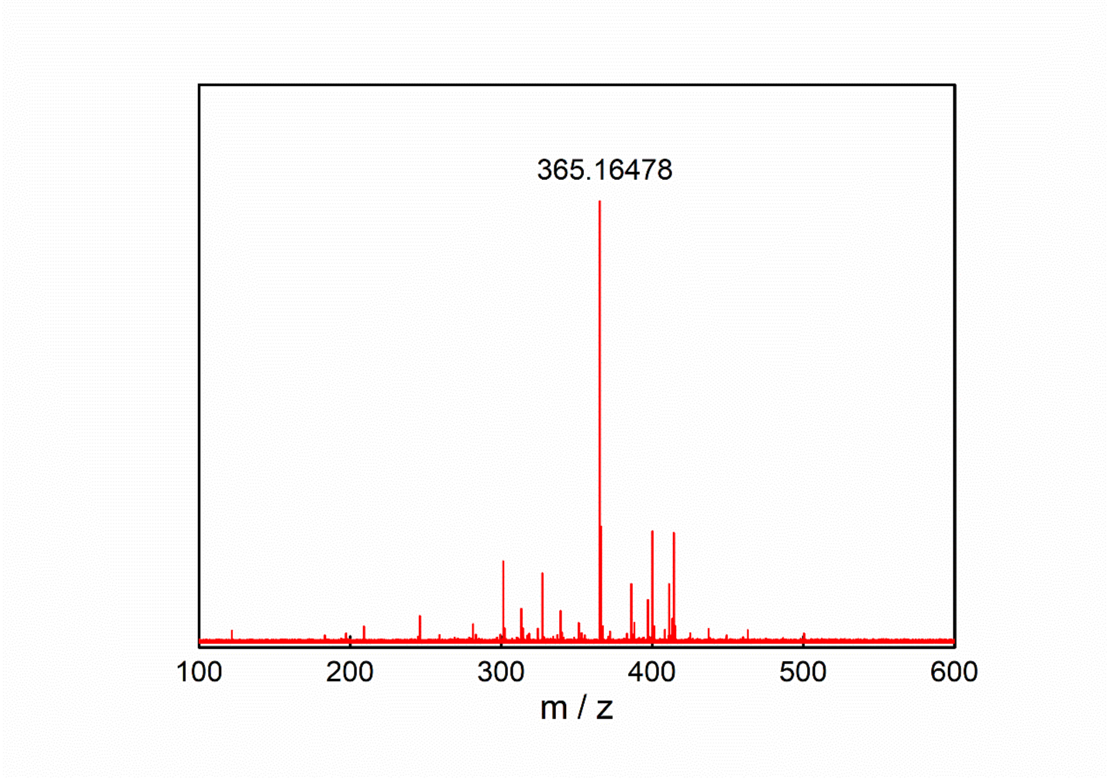

Supplementary Figure 11. ESI-HRMS spectrum of **G1**.

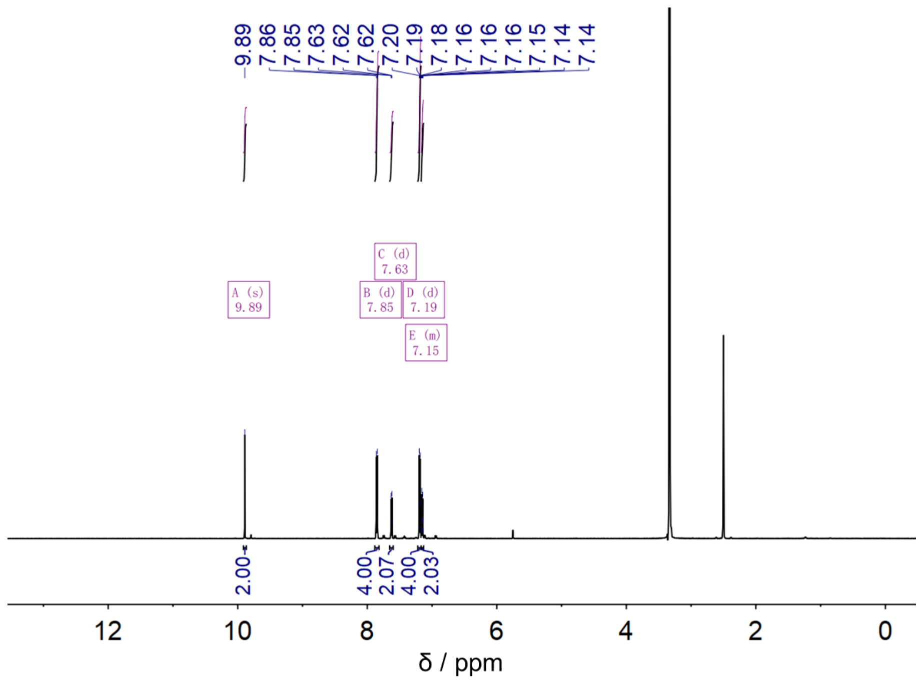

Supplementary Figure 12. ^{1}H NMR spectrum of **2a** (600 MHz, 298 K, DMSO-d_6).

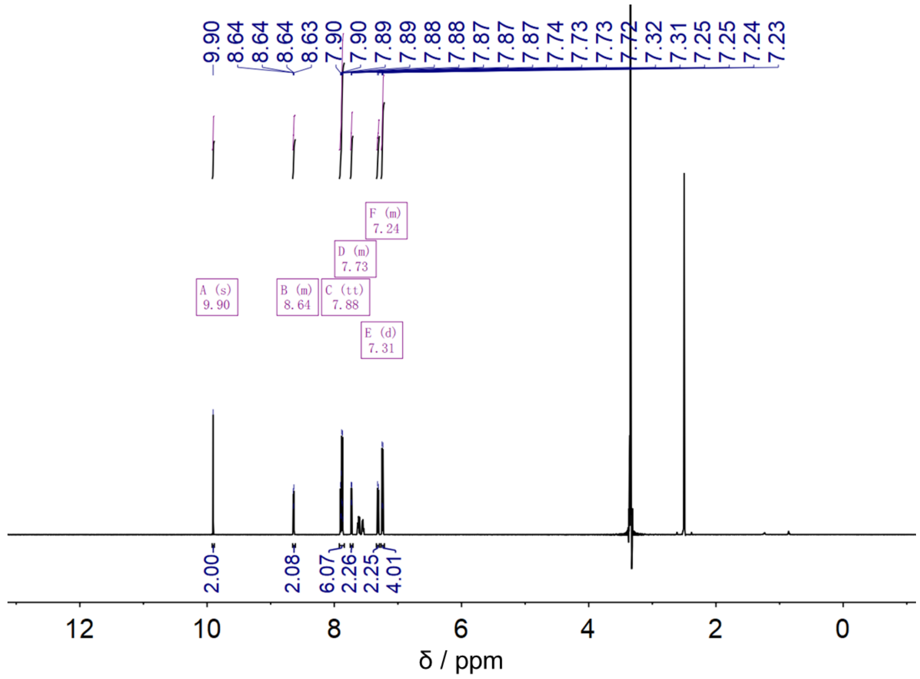

Supplementary Figure 13. ^{1}H NMR spectrum of **2b** (600 MHz, 298 K, DMSO-d_6).

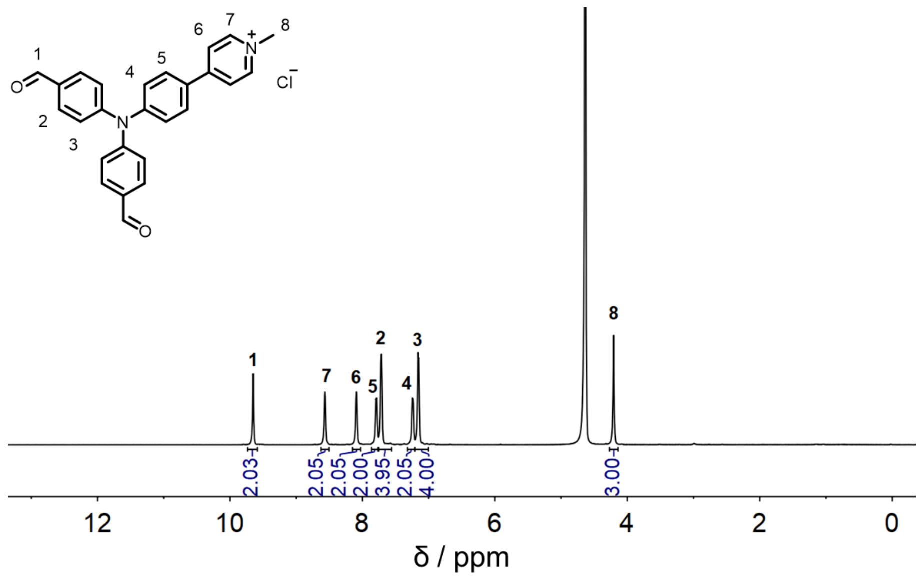

Supplementary Figure 14. ^{1}H NMR spectrum of **G2** (600 MHz, 298 K, D_2O).

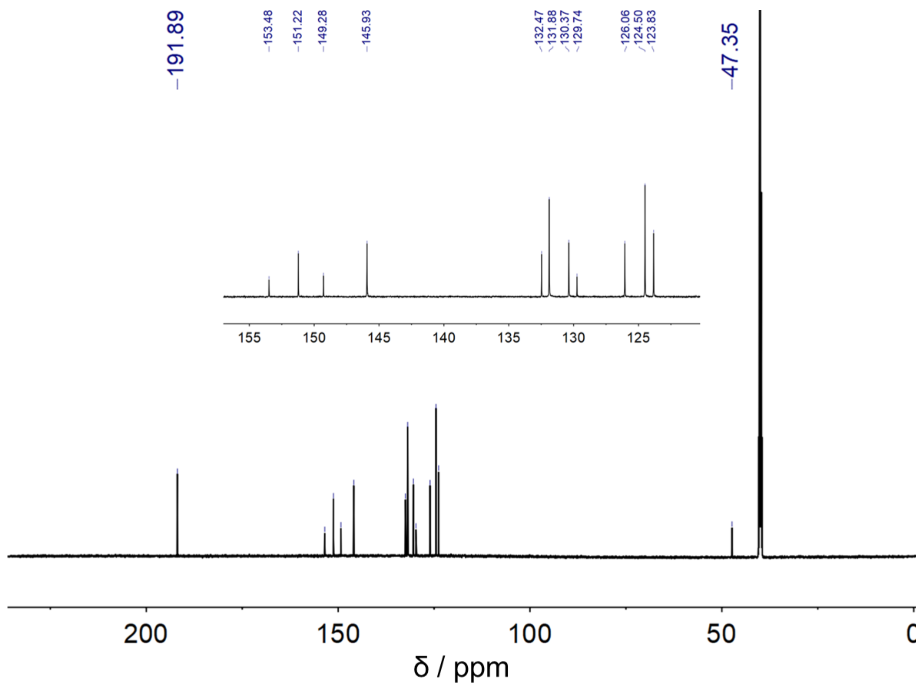

Supplementary Figure 15. ^{13}C NMR spectrum of **G2** (150 MHz, 298 K, DMSO-d_6).

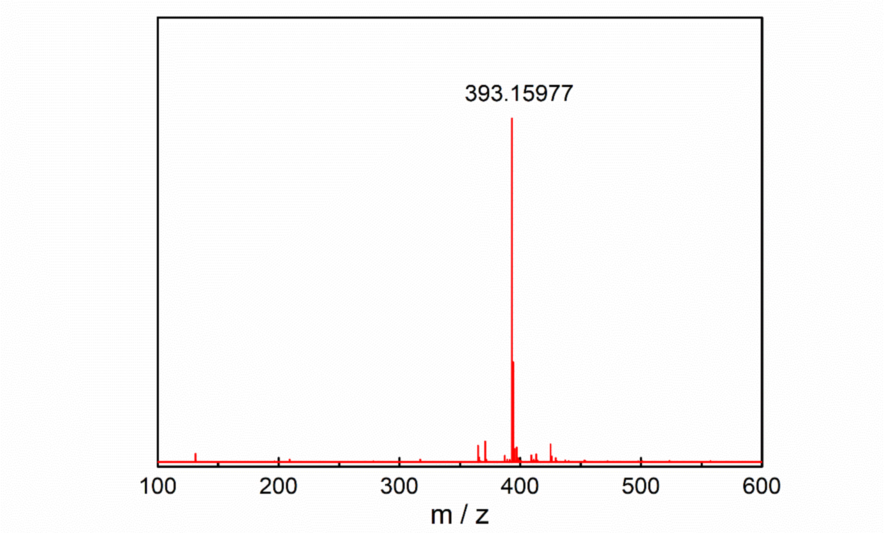

Supplementary Figure 16. ESI-HRMS spectrum of **G2**.

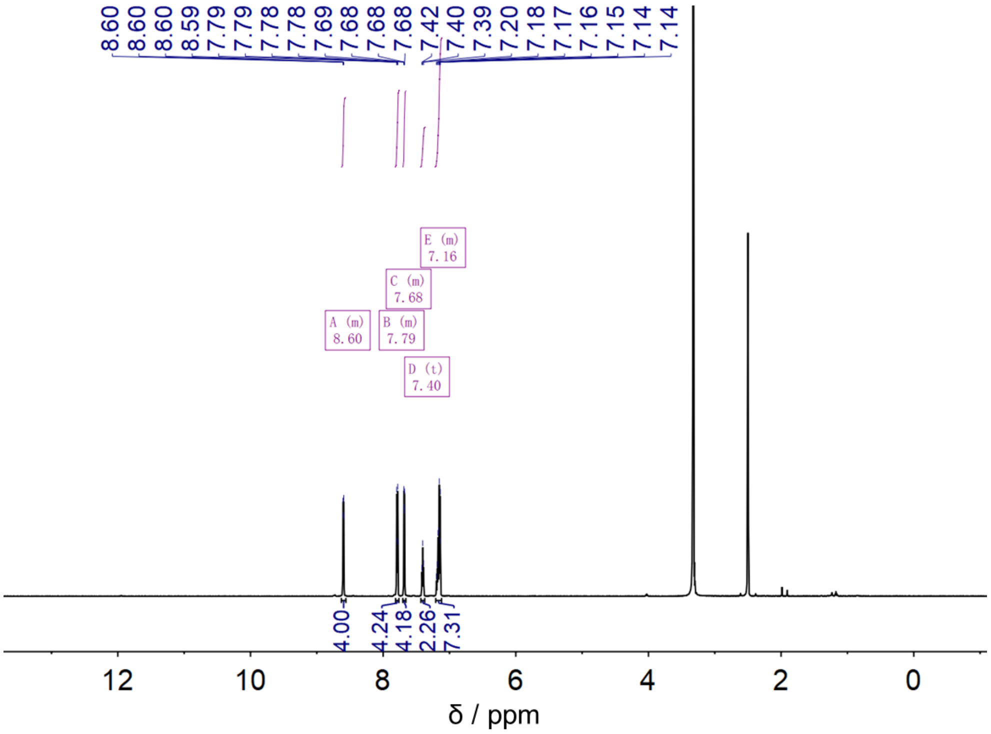

Supplementary Figure 17. ^{1}H NMR spectrum of **3a** (600 MHz, 298 K, DMSO-d_6).

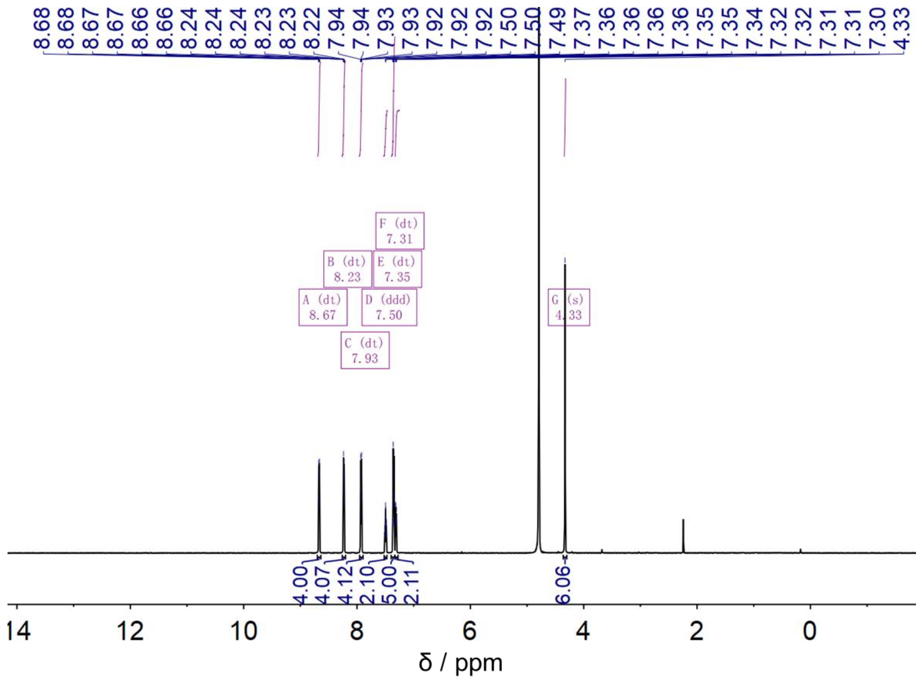

Supplementary Figure 18. ^{1}H NMR spectrum of **G3** (600 MHz, 298 K, D_2O).

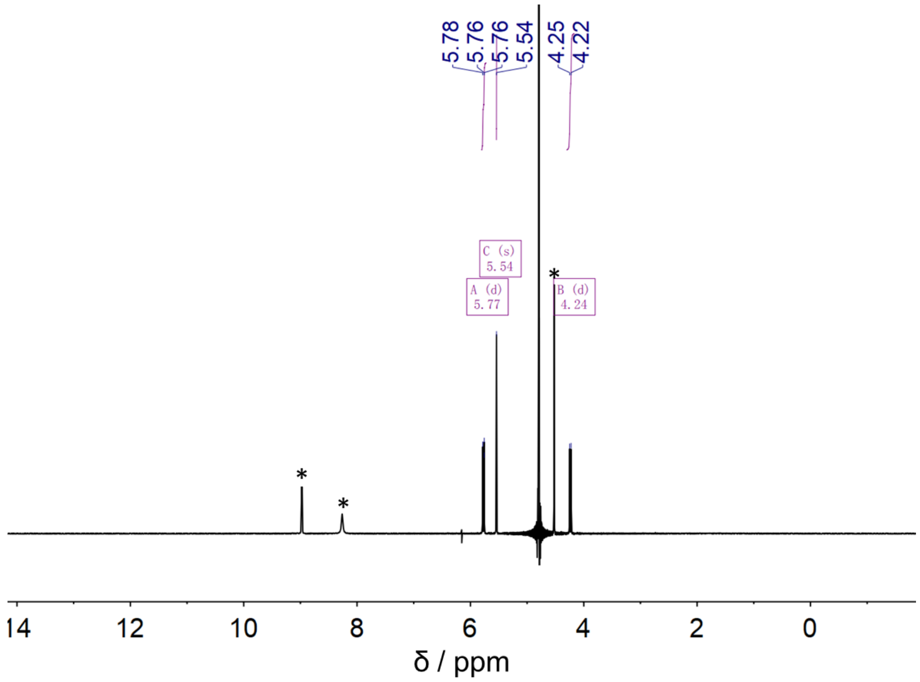

Supplementary Figure 19. ^{1}H NMR spectrum of CB[8] (600 MHz, 298 K, D_2O) with added methylviologen (the proton signals of methylviologen are marked with "*"). Methylviologen was added to improve the solubility of CB[8].

Fluorescence spectra of G1 in different solvents

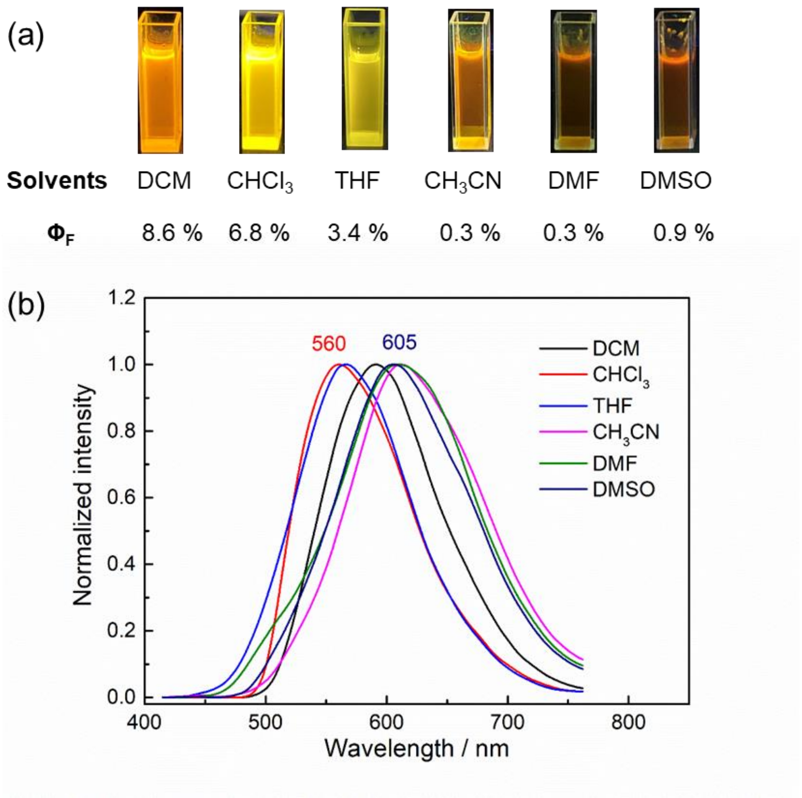

Supplementary Figure 20. (a) Photographs of **G1** in different solvents under the excitation of 365 nm UV light (the fluorescence quantum yields of **G1** in different solvents are presented). (b) Normalized emission spectra of **G1** in different solvents (λ_{ex} = 400 nm).

MCL behavior of G1-G3

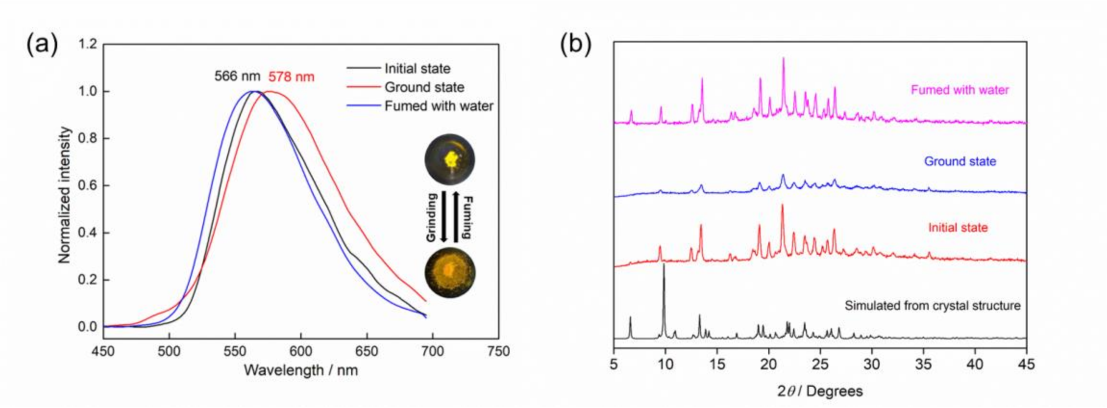

Supplementary Figure 21. (a) PL spectra of **G1** powder under different treatments (λ_{ex} = 420 nm). (b) Powder X-ray diffraction patterns of **G1** powder under different treatments.

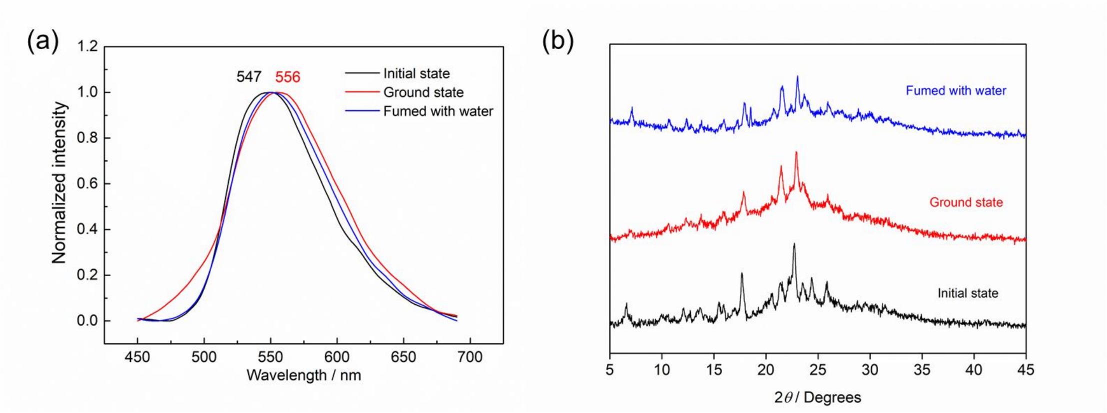

Supplementary Figure 22. (a) PL spectra of **G2** powder under different treatments (λ_{ex} = 420 nm). (b) Powder X-ray diffraction patterns of **G2** powder under different treatments.

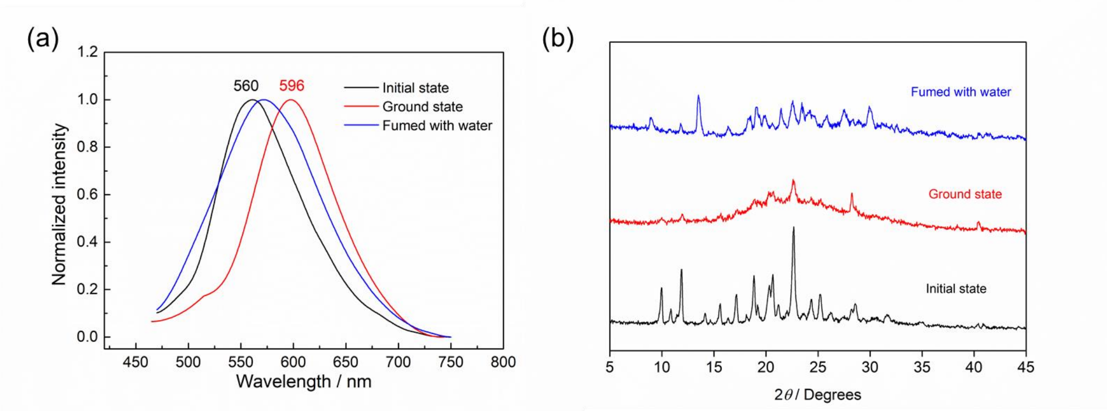

Supplementary Figure 23. (a) PL spectra of **G3** powder under different treatments (λ_{ex} = 420 nm). (b) Powder X-ray diffraction patterns of **G3** powder under different treatments.

Fluorescence lifetime decay profiles

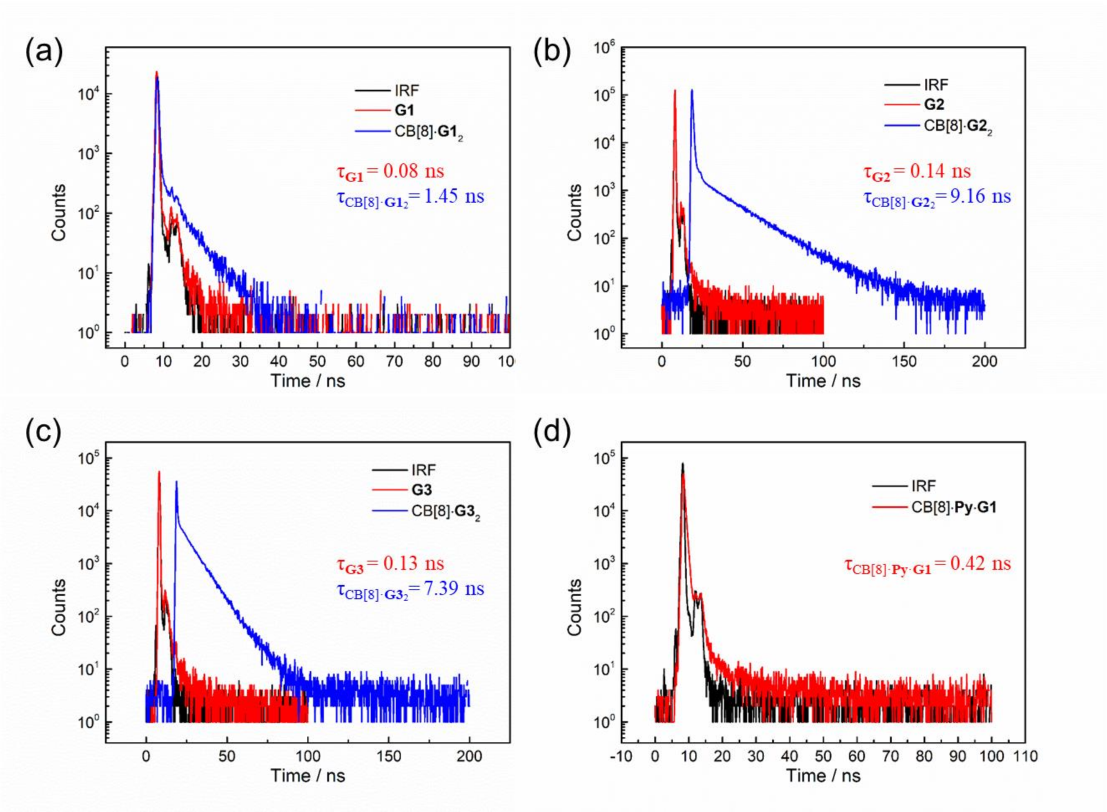

Supplementary Figure 24. Fluorescence lifetime decay profiles of (a) **G1** and CB[8]·**G1**$_2$, (b) **G2** and CB[8]·**G2**$_2$, (c) **G3** and CB[8]·**G3**$_2$, and (d) CB[8]·**Py**·**G1** in aqueous solution. The concentration of all samples is 2.0×10^{-5} M.

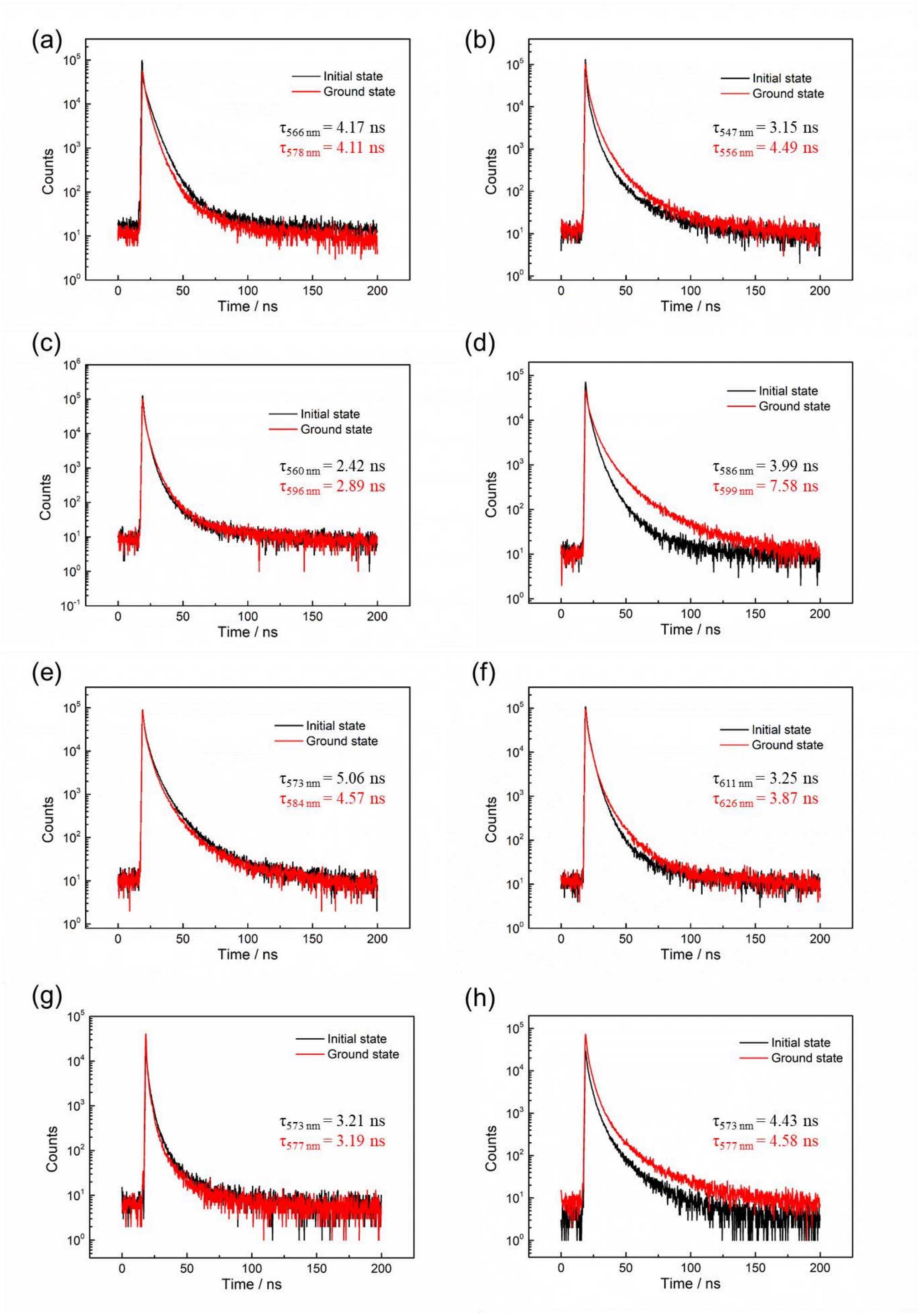

Supplementary Figure 25. Fluorescence lifetime decay profiles of (a) **G1**, (b) **G2**, (c) **G3**, (d) CB[8]·**G1**$_2$, (e) CB[8]·**G2**$_2$, (f) CB[8]·**G3**$_2$ (g) **Py**·**G1** and (h) CB[8]·**Py**·**G1** in solid state under different treatments.

ITC data for G1 with CB[8]

Supplementary Table 1. Thermodynamic data for compound **G1** binding with CB[8].

	K_a (M^{-1})	ΔG (kJ/mol)[a]	ΔH (kJ/mol)	ΔS (J/mol·K)
Step 1	2.130×10^7	-41.83	-36.90	16.54
Step 2	3.758×10^5	-31.82	-35.31	-11.71

[a] The standard free energy (ΔG) can be obtained according to the following equation:

$$\Delta G = \Delta H - T\Delta S$$

where T is the absolute temperature.

Host-guest complexation of CB[8] and G2/G3

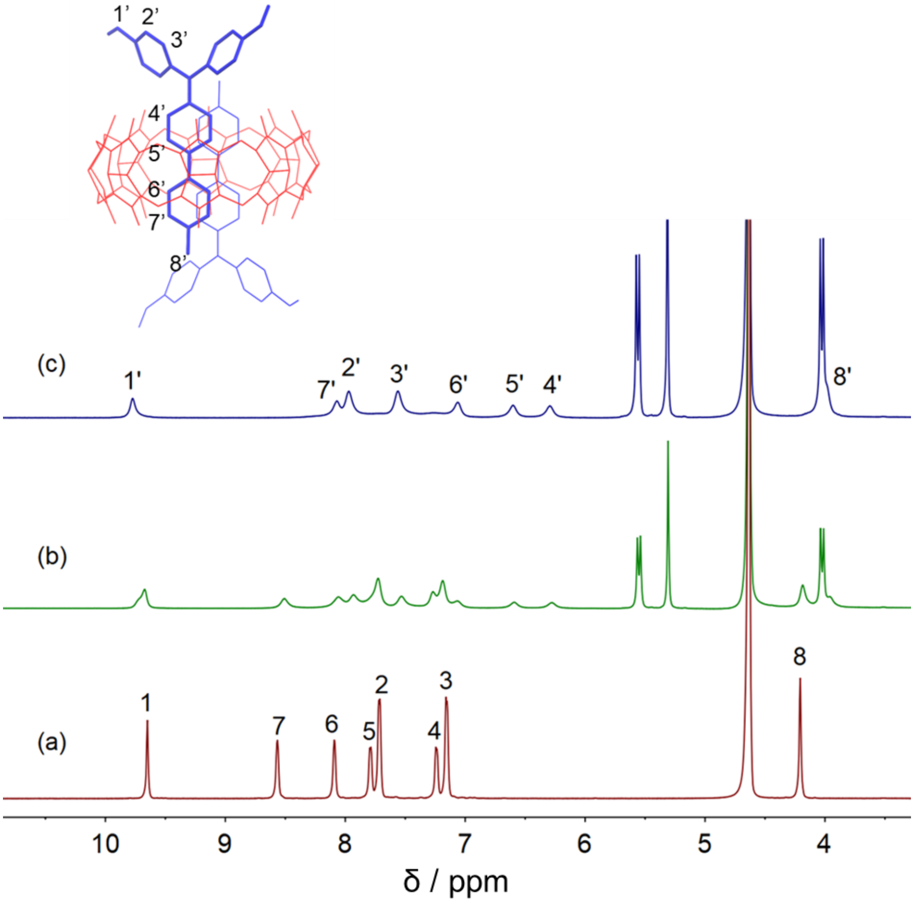

Supplementary Figure 26. ^{1}H NMR spectra of **G2** (2.0 mM, 600 MHz, D_2O, 298 K) with (a) 0 equiv. of CB[8], (b) 0.25 equiv. of CB[8], (c) 0.5 equiv. of CB[8].

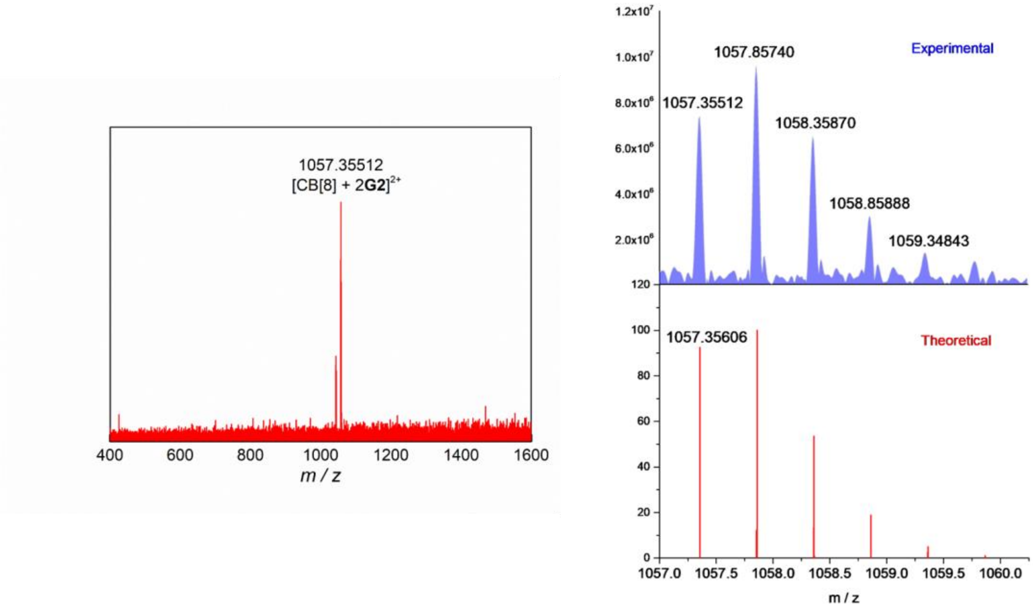

Supplementary Figure 27. ESI-HRMS spectrum of CB[8]·**G2**$_2$.

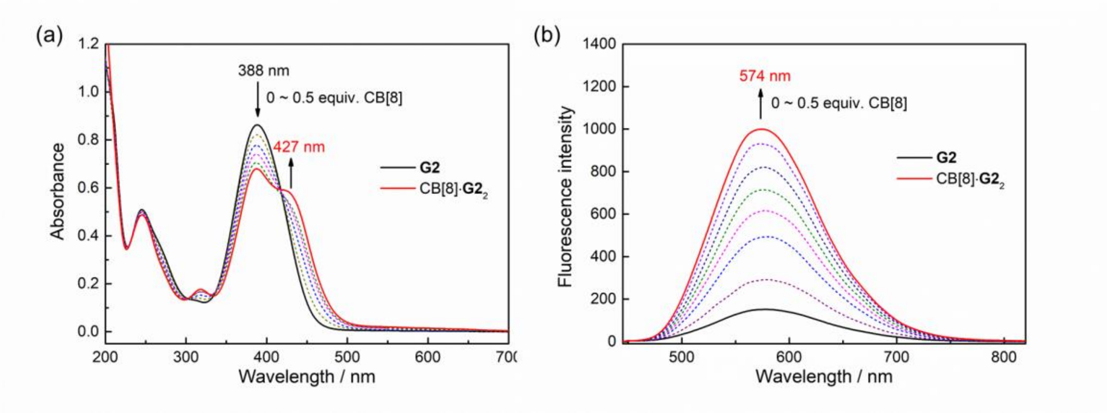

Supplementary Figure 28. UV-vis absorption spectra (a) and fluorescence spectra (b) of **G2** (20 μM in H_2O, 298 K) with different equiv. of CB[8] (0 ~ 0.5 equiv.) (λ_{ex} = 420 nm).

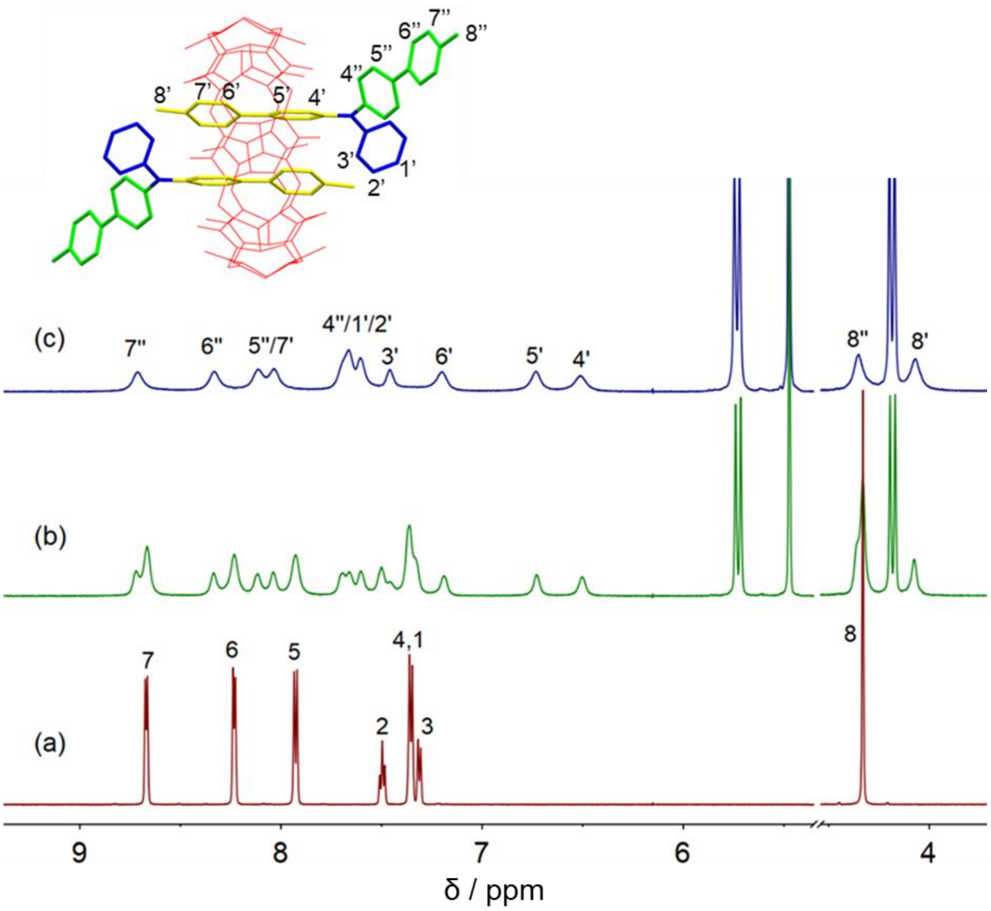

Supplementary Figure 29. ^{1}H NMR spectra of **G3** (2.0 mM, 600 MHz, D_2O, 298 K) with 0 equiv. of CB[8] (a), 0.25 equiv. of CB[8] (b), 0.50 equiv. of CB[8] (c). Characters 4'−8' and 4''−8'' represent the resonance signals of 4-phenylpyridine groups which located inside and outside the cavity of CB[8], respectively.

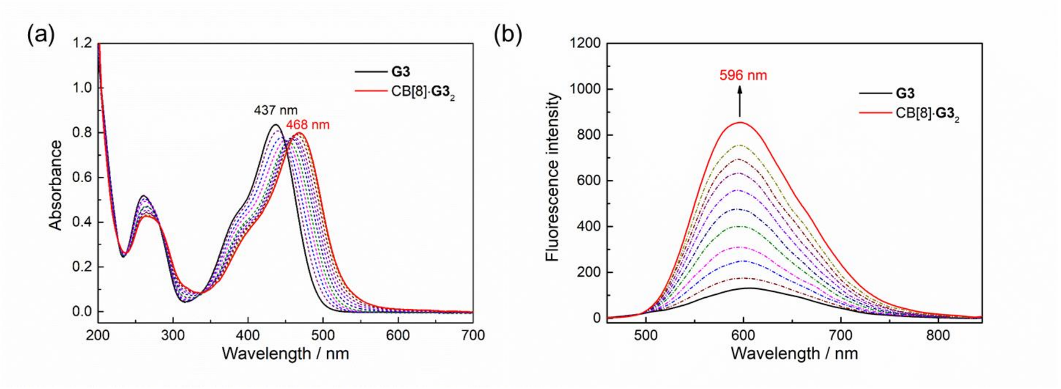

Supplementary Figure 30. UV-vis absorption spectra (a) and fluorescence spectra (b) of **G3** (20 μM in H_2O, 298 K) with addition of different equiv. of CB[8] (0 ~ 0.5 equiv.) (λ_{ex} = 420 nm).

MCL behavior of CB[8]·G$_2$

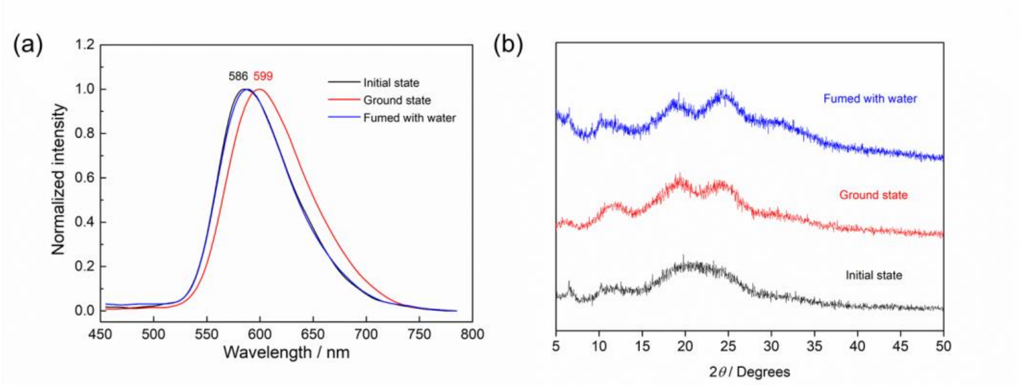

Supplementary Figure 31. (a) PL spectra of CB[8]·**G1**$_2$ powder under different treatments (λ_{ex} = 420 nm). (b) Powder X-ray diffraction patterns of CB[8]·**G1**$_2$ powder under different treatments.

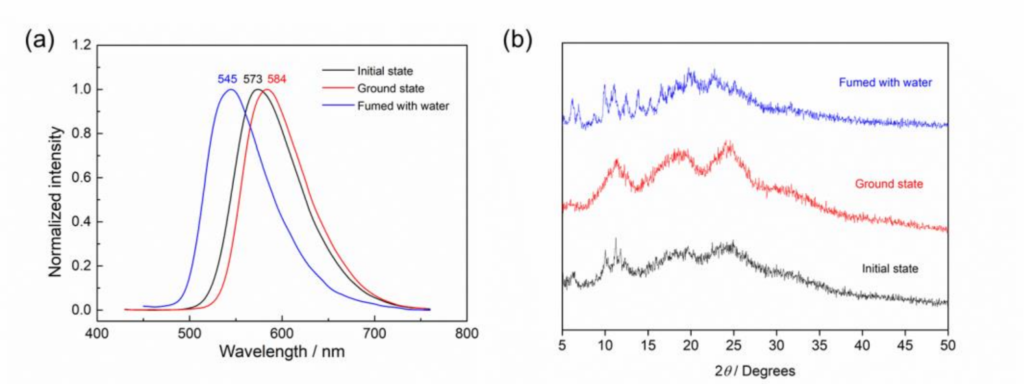

Supplementary Figure 32. (a) PL spectra of CB[8]·**G2**$_2$ powder under different treatments (λ_{ex} = 420 nm). (b) Powder X-ray diffraction patterns of CB[8]·**G2**$_2$ powder under different treatments.

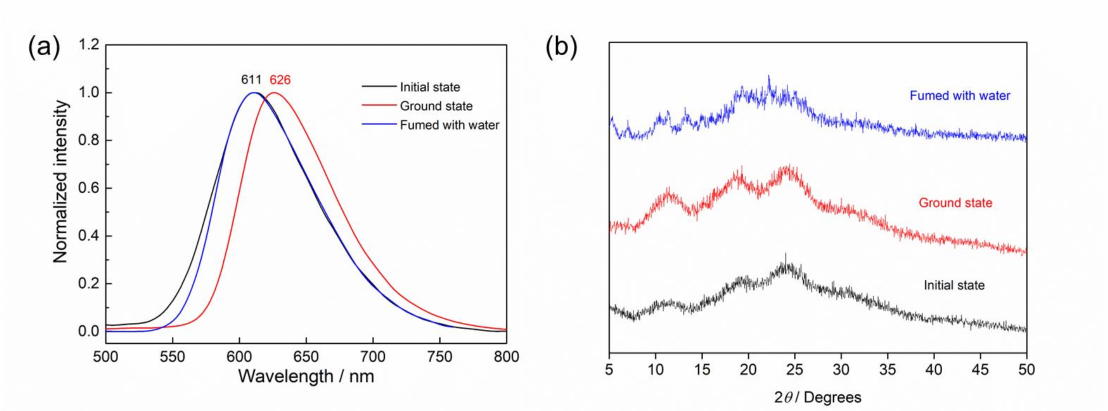

Supplementary Figure 33. (a) PL spectra of CB[8]·**G3**$_2$ powder under different treatments (λ_{ex} = 420 nm). (b) Powder X-ray diffraction patterns of CB[8]·**G3**$_2$ powder under different treatments.

Crystal structure analysis

Supplementary Table 2. Dihedral angles θ_{1-3} of adjacent (hetero)aryl groups in **G1**-involved crystals.

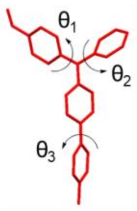

G1

Compound	**G1**		CB[8]·**G1**$_2$				CB[8]·**Py**·**G1**	
Conformation	a	b	c	d	e	f	g	h
θ_1	75.22	69.36	55.61	50.80	54.34	52.77	62.75	61.85
θ_2	86.00	83.12	59.86	69.32	70.34	66.18	61.43	78.64
θ_3	26.21	31.65	5.09	7.77	23.93	23.49	4.92	24.36
sum	187.43	184.13	120.56	127.89	148.61	142.44	129.10	164.85

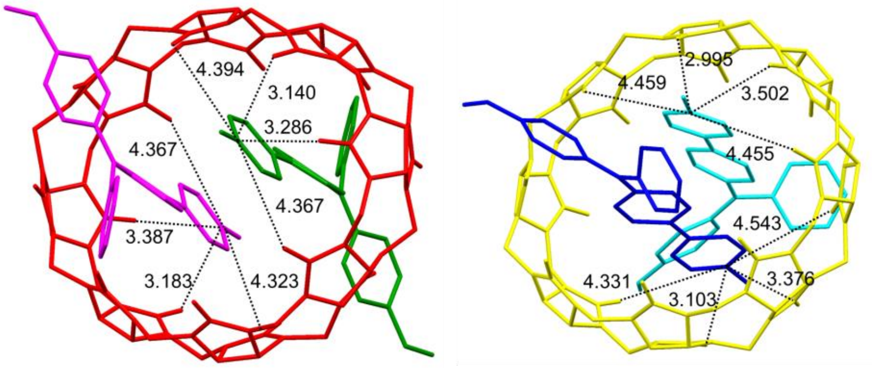

Supplementary Figure 34. Ion-dipole interactions in CB[8]·**G1**$_2$ crystal (the distance between N^+ of **G1** and C=O of CB[8] are presented, counterions are omitted for clarity).

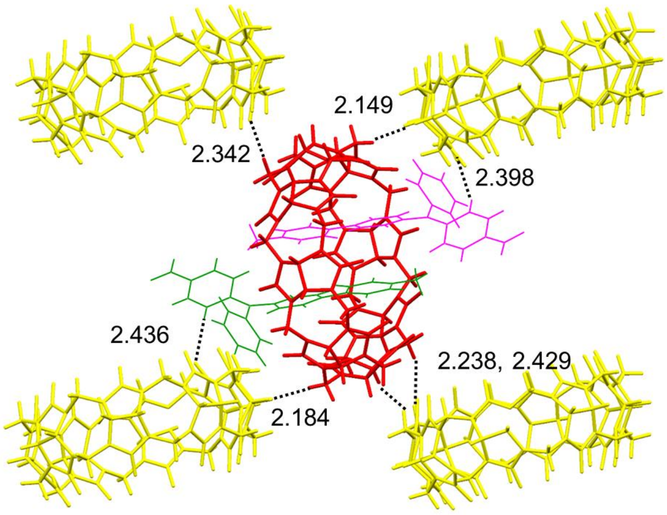

Supplementary Figure 35. Multiple C−H···O interactions between CB[8]·**G1**$_2$ and adjacent CB[8] molecules (counterions are omitted for clarity).

Solid state absorption spectra of G1 and CB[8]·G1$_2$

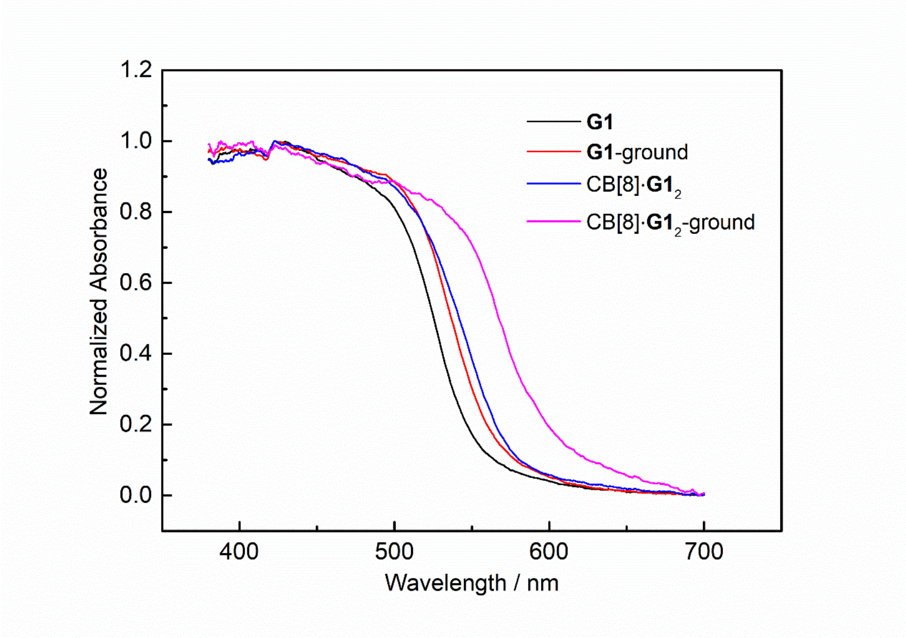

Supplementary Figure 36. Solid state absorption spectra for the as-prepared and ground samples of **G1** and CB[8]·**G1**$_2$.

Host-guest recognition of CB[8] and Py

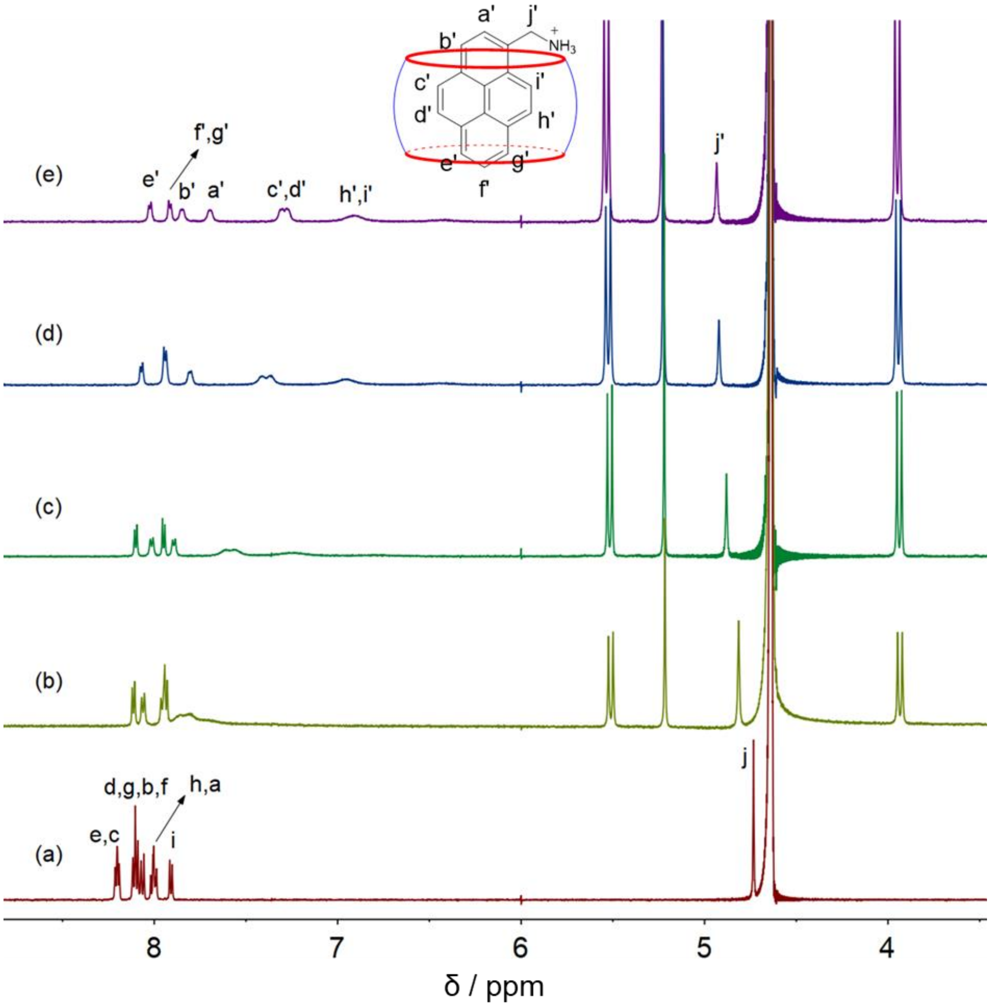

Supplementary Figure 37. ^{1}H NMR spectra of **Py** (1.0 mM, 600 MHz, D_2O, 298 K) with (a) 0 equiv. of CB[8], (b) 0.25 equiv. of CB[8], (c) 0.5 equiv. of CB[8], (d) 0.75 equiv. of CB[8] and (e) 1.00 equiv. of CB[8].

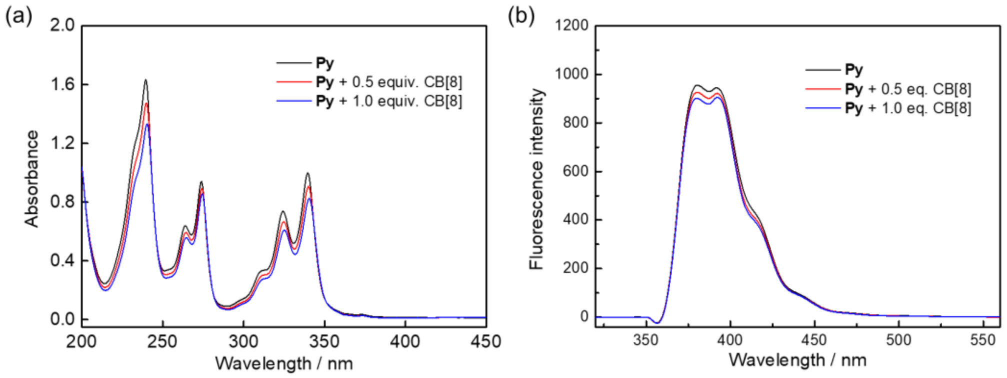

Supplementary Figure 38. UV-vis absorption spectra (a) and fluorescence spectra (b) of **Py** (20 μM in H_2O, 298 K) with different equiv. of CB[8] (0 ~ 1.0 equiv.) (λ_{ex} = 310 nm).

Host-guest recognition and MCL behavior of CB[8]·Py·G1

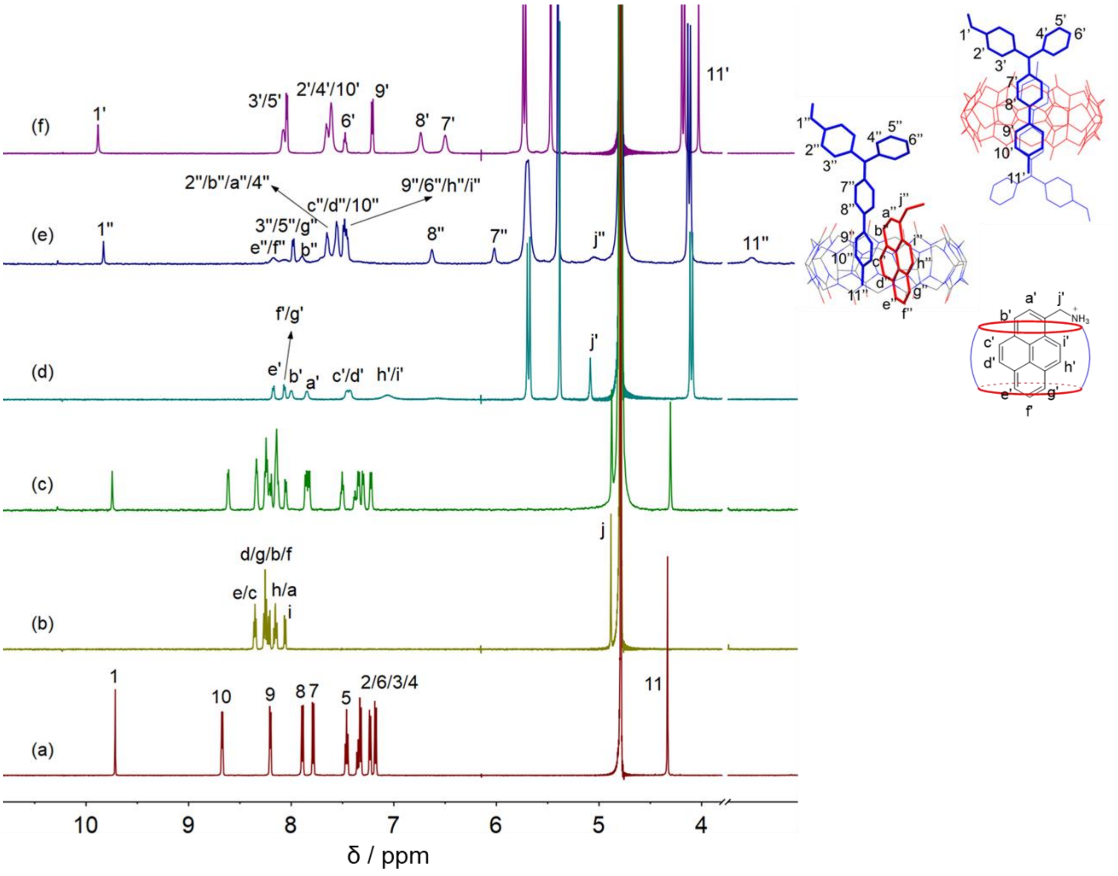

Supplementary Figure 39. ^{1}H NMR spectra (600 MHz, D_2O, 298 K) of **G1** (a), **Py** (b), **G1** + **Py** (1:1) (c), CB[8]·**Py** (d), CB[8]·**Py**·**G1** (e), and CB[8]·**G1**$_2$ (f). The concentration of CB[8], **Py** and **G1** in all sample is 1.0 mM. Characters 1−11, a−j represent the resonance signals of **G1** and **Py** as free. Characters 1'−11', a'−j' represent the resonance signals of **G1** and **Py** in CB[8]·**G1**$_2$ and CB[8]·**Py**, respectively. Characters 1''−11'', a''−j'' represent the resonance signals of **G1** and **Py** in CB[8]·**Py**·**G1**.

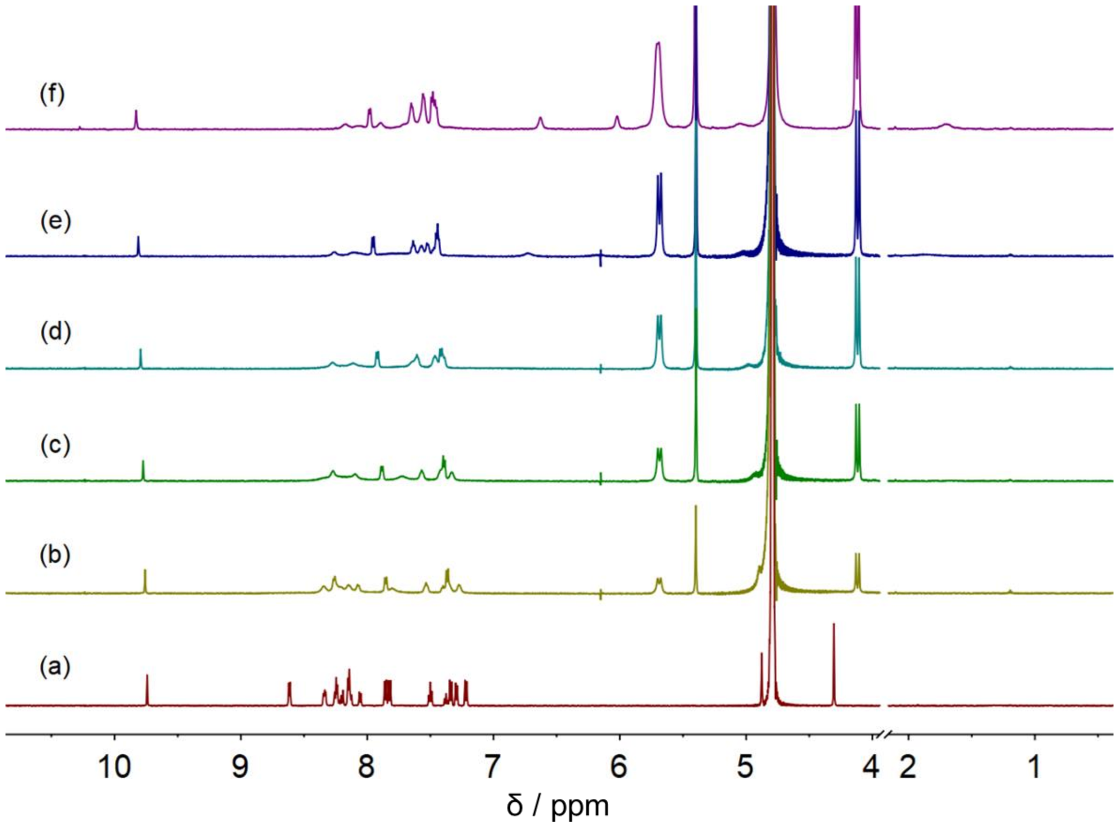

Supplementary Figure 40. ^1H NMR spectra of **Py** + **G1** (1:1, 1.0 mM, 600 MHz, D_2O, 298 K) (a) with 0.2 equiv. of CB[8] (b), 0.4 equiv. of CB[8] (c), 0.6 equiv. of CB[8] (d), 0.8 equiv. of CB[8] (e), 1.0 equiv. of CB[8] (f).

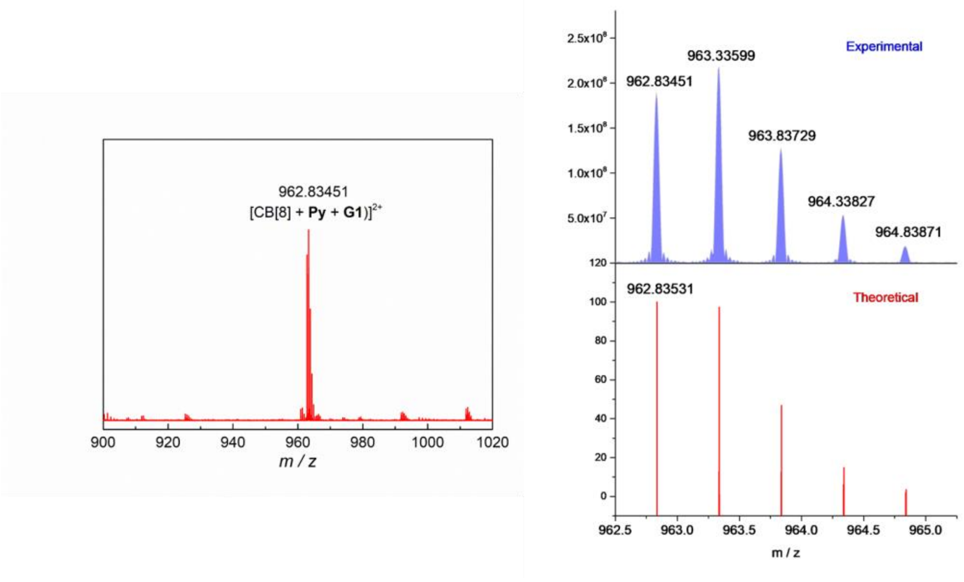

Supplementary Figure 41. ESI-HRMS spectrum of CB[8]·**Py**·**G1**.

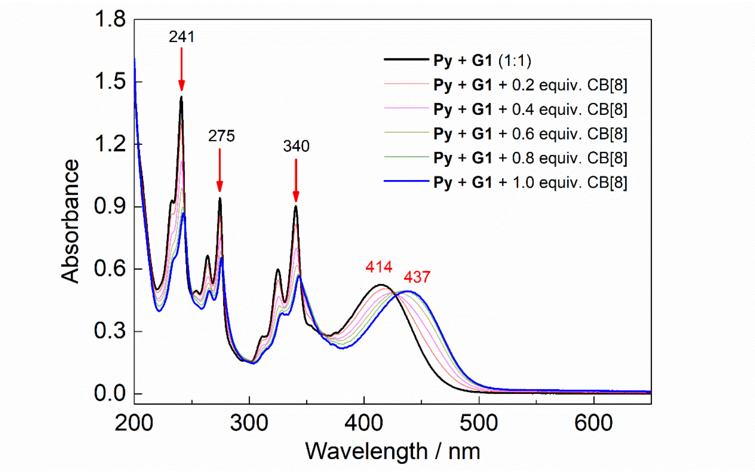

Supplementary Figure 42. UV-vis absorption spectra of **Py** + **G1** (1:1, 20 μM in H_2O, 298 K) with different equiv. of CB[8] (0 ~ 1.0 equiv.).

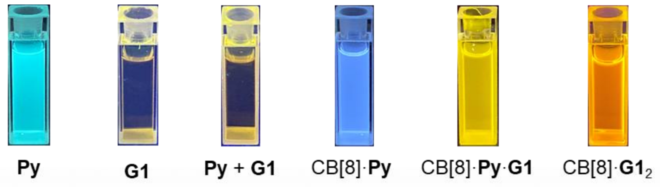

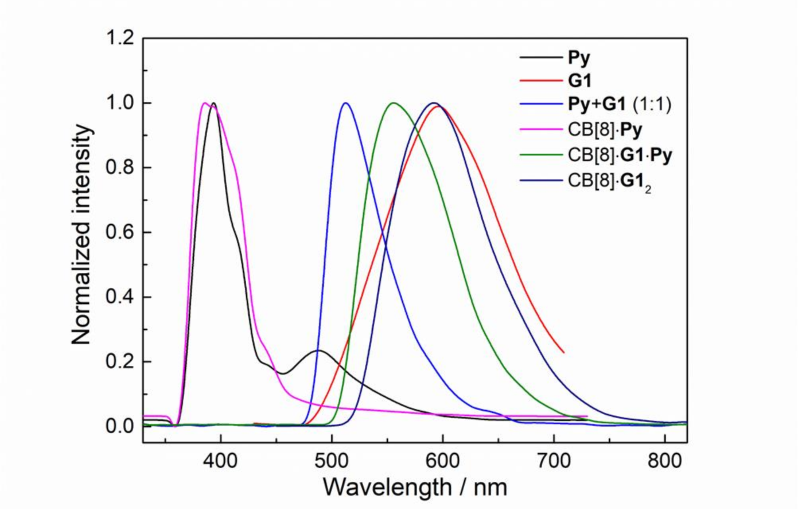

Supplementary Figure 43. Normalized emission spectra of **Py**, **G1**, **Py**+**G1** (1:1), CB[8]·**Py**, CB[8]·**Py**·**G1** and CB[8]·$\mathbf{G1}_2$ in aqueous solution (1.0 mM, 298 K). The excitation wavelength for **Py**, CB[8]·**Py** and **Py**+**G1** (1:1) is 340 nm, and the excitation wavelength for **G1**, CB[8]·$\mathbf{G1}_2$ and CB[8]·**Py**·**G1** is 420 nm. Insert: photos of **Py**, **G1**, **Py**+**G1** (1:1), CB[8]·**Py**, CB[8]·**Py**·**G1** and CB[8]·$\mathbf{G1}_2$ in aqueous solution under excitation of 365 nm UV light (1.0 mM, 298 K). Notes: (1) The green emission of **Py** at 488 nm at high concentration (1.0 mM) is assigned to the excimer emission of pyrene; the 1:1 complexation between **Py** and CB[8] hinders the formation of excimer, so that CB[8]·**Py** exhibits the monomer emission at 394 nm. (2) The emissions of **G1** and **Py**+**G1** (1:1) are much weaker than other samples, so we use the normalized spectra to illustrate the change of emission wavelength.

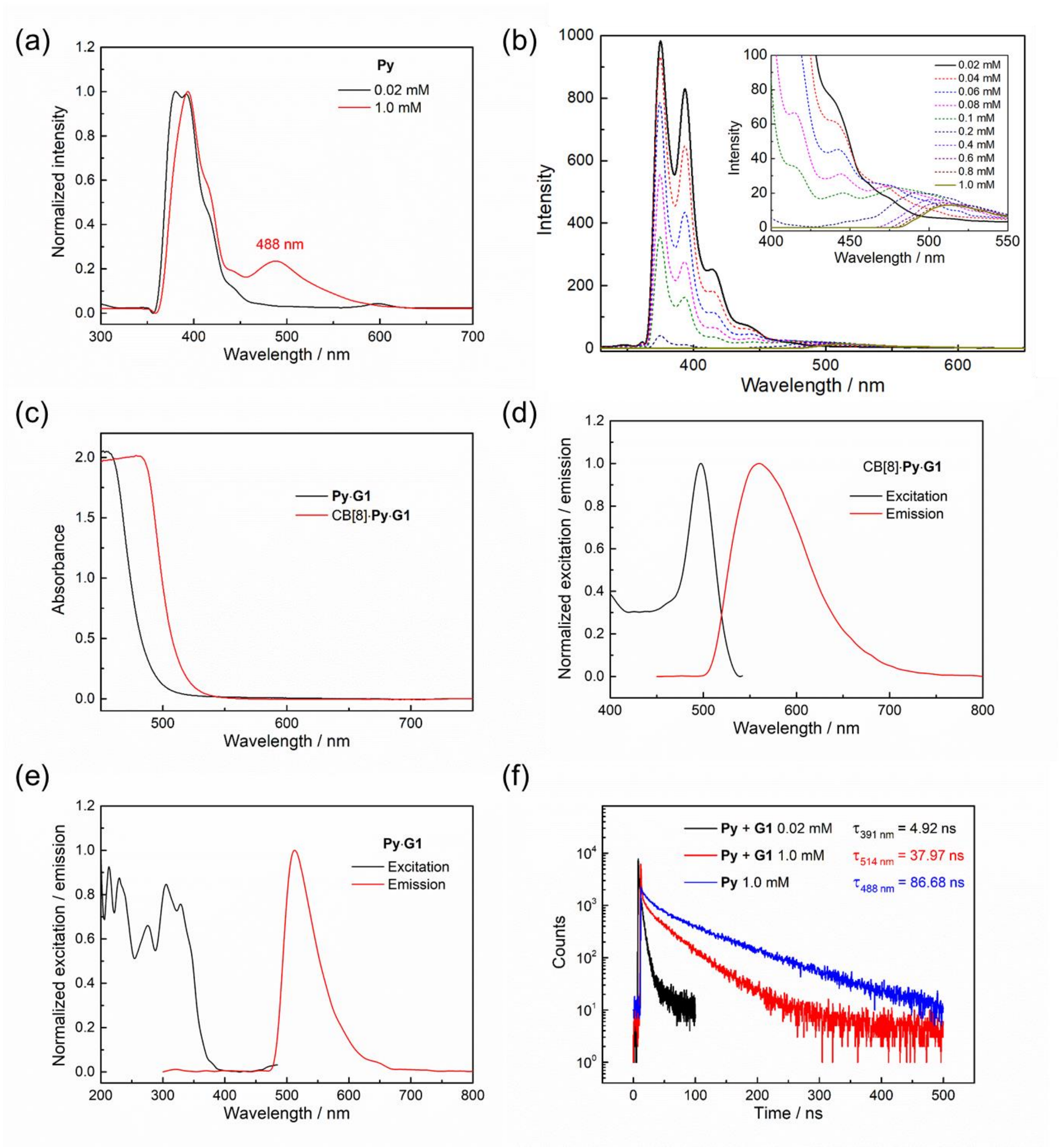

Supplementary Figure 44. (a) Normalized emission spectra of **Py** at different concentrations (λ_{ex} = 340 nm). (b) Fluorescence spectra of **Py**+**G1** (1:1) at different concentrations (λ_{ex} = 340 nm, insert: emission intensity of **Py**+**G1** (1:1) at different concentrations in the range of 400-550 nm). (c) UV-vis absorption spectra of **Py**+**G1** (1:1) and CB[8]·**Py**·**G1** (0.5 mM). (d) Normalized excitation and emission spectra of CB[8]·**Py**·**G1** (0.5 mM, the excitation spectrum is collected by monitoring the emission at 560 nm). (e) Normalized excitation and emission spectra of **Py**·**G1** (1.0 mM, the excitation spectrum is collected by monitoring the emission at 514 nm). (f) Fluorescence emission lifetime of **Py** and **Py**+**G1** (1:1) at different concentration. All of these data were collected in water at 298 K.

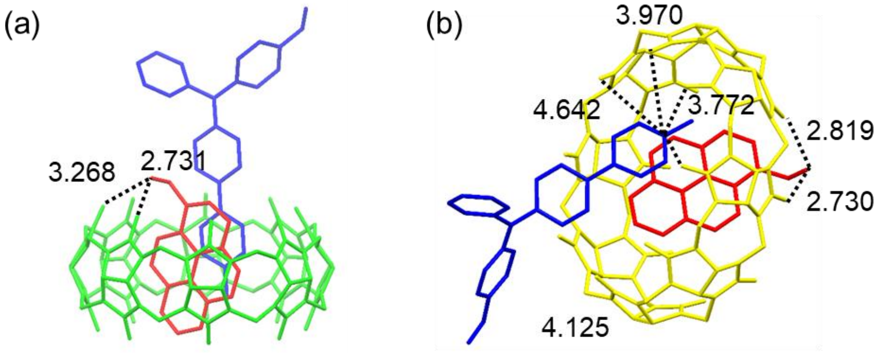

Supplementary Figure 45. Ion-dipole interactions in (CB[8]·**Py**·**G1**)-a (a) and (CB[8]·**Py**·**G1**)-b (b) (the distance between N$^+$ of **G1**, **Py** and C=O of CB[8] are presented, counterions and hydrogen atoms are omitted for clarity).

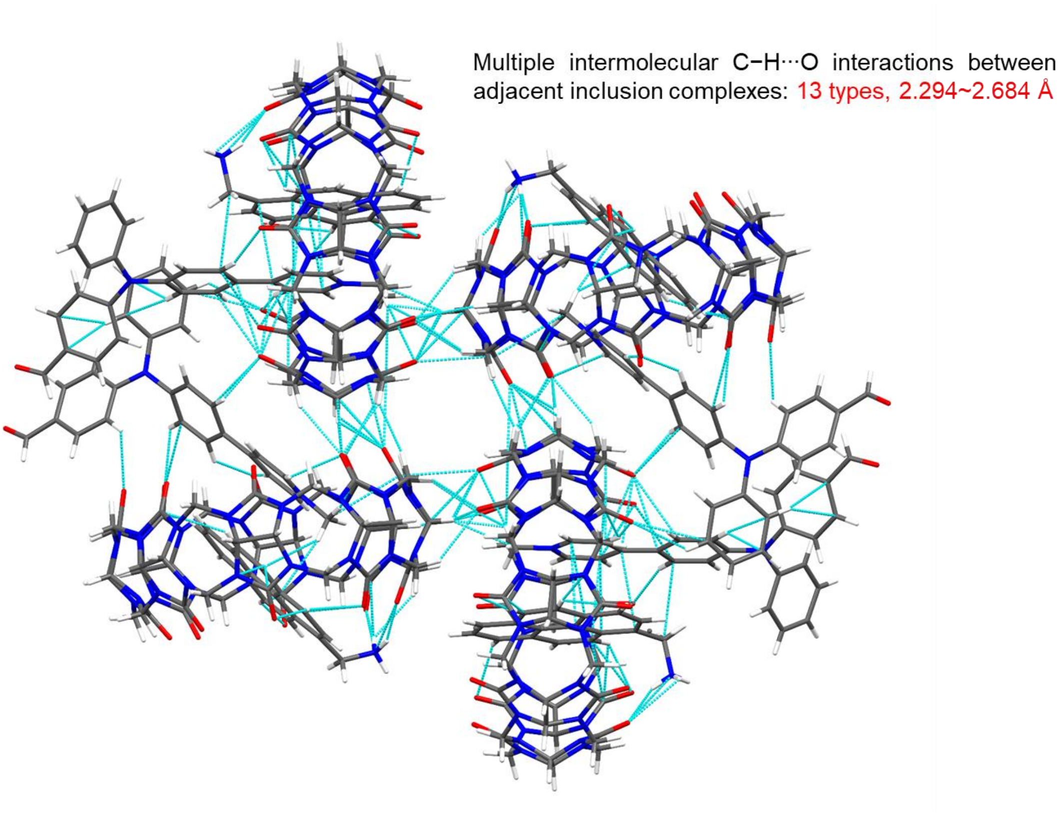

Supplementary Figure 46. Multiple intermolecular C−H···O interactions in CB[8]·**Py**·**G1** crystal (counterions are omitted for clarity).

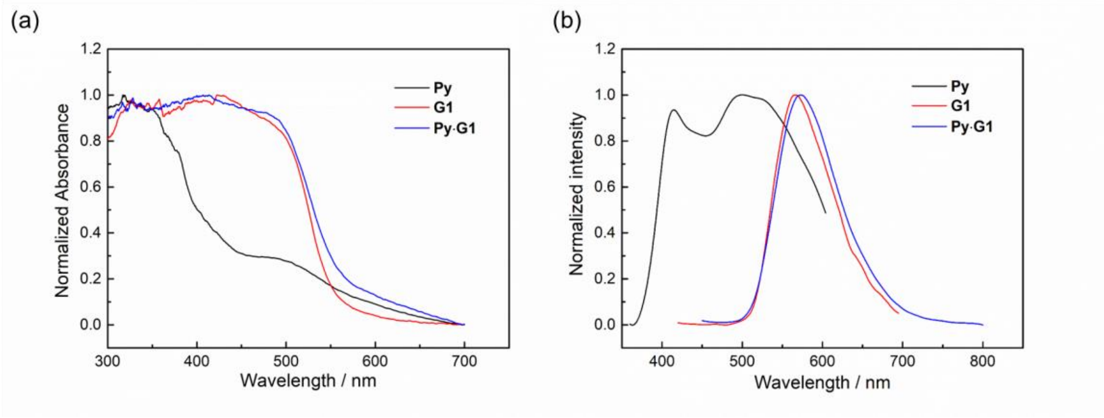

Supplementary Figure 47. Normalized absorption (a) and fluorescence emission spectra (b) of **Py**, **G1** and **Py·G1** in solid state (λ_{ex} (**Py**) = 340 nm, λ_{ex} (**G1** and **Py·G1**) = 340 nm).

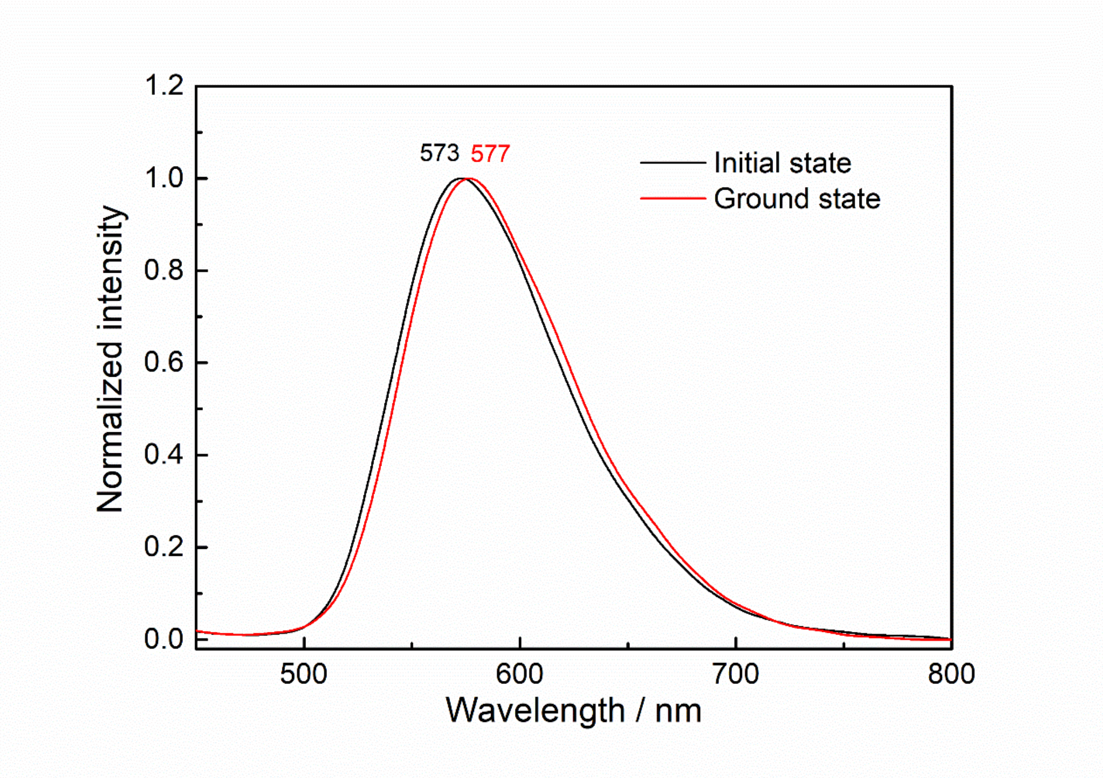

Supplementary Figure 48. PL spectra of **Py** + **G1** (1:1) powder under different treatments (λ_{ex} = 420 nm).

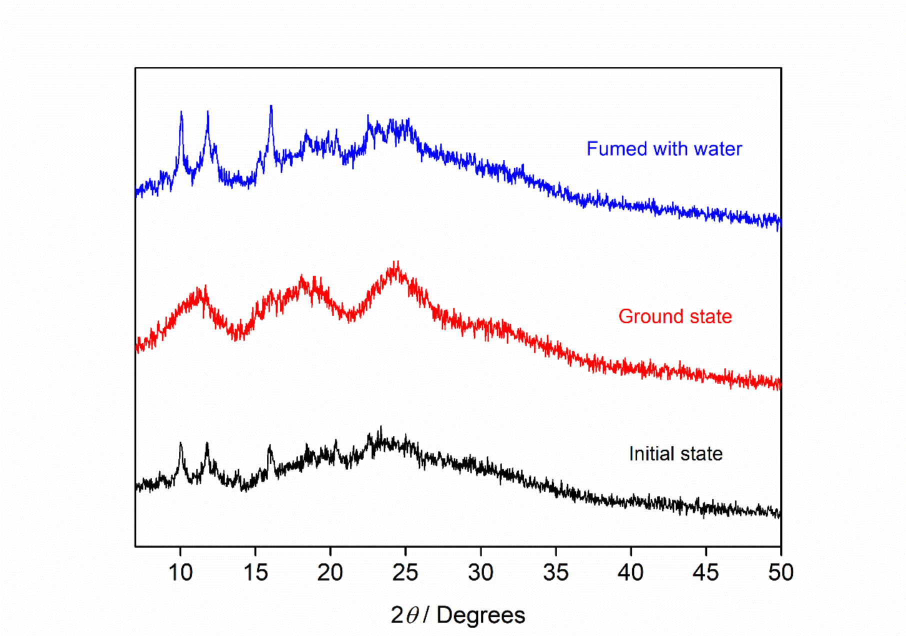

Supplementary Figure 49. Powder X-ray diffraction patterns of CB[8]·**Py**·**G1** powder under different treatments.

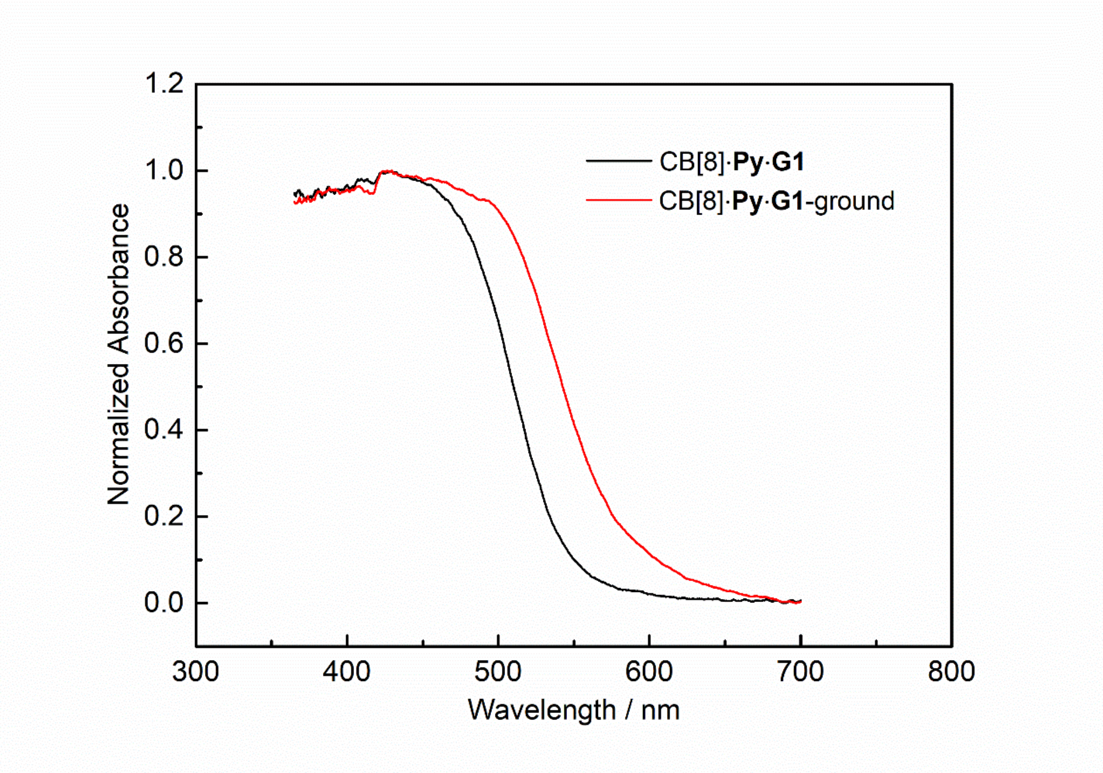

Supplementary Figure 50. Solid state absorption spectra for the as-prepared and ground samples of CB[8]·**Py**·**G1**.

Crystal data

Supplementary Table 3. Crystal data and structure refinement for **G1**.

Compound	**G1**
CCDC	2159417
Empirical formula	$C_{25}H_{21}ClN_2O$
Formula weight	400.89
Temperature/K	115.00
Crystal system	triclinic
Space group	*P*-1
a/Å	9.1291(12)
b/Å	9.5154(13)
c/Å	27.370(4)
α/°	82.110(4)
β/°	80.610(5)
γ/°	86.352(4)
Volume/Å^3	2321.5(5)
Z	4
ρ_{calc} g/cm^3	1.147
μ/mm^{-1}	1.037
F(000)	840.0
Crystal size/mm^3	0.16× 0.16× 0.10
Radiation	GaKα (λ = 1.34139)
2Θ range for data collection/°	5.742 to 110.356
Reflections collected	8819
Data/restraints/parameters	8819/0/525
Goodness-of-fit on F^2	1.001
Final R indexes [I>=2σ (I)]	R_1 = 0.1013, wR_2 = 0.2236
Final R indexes [all data]	R_1 = 0.1265, wR_2 = 0.2340
Largest diff. peak/hole / e Å^{-3}	0.44/-0.70

Supplementary Table 4. Crystal data and structure refinement for CB[8]·**G1**$_2$ and CB[8]·**G2**$_2$.

Compound	CB[8]·**G1**$_2$	CB[8]·**G2**$_2$
CCDC	2201842	2201844
Empirical formula	$C_{98}H_{90}Cl_2N_{36}O_{18}$	$C_{100}H_{90}Cl_2N_{36}O_{20}$
Formula weight	2130.95	2186.97
Temperature/K	200.00	200.0
Crystal system	monoclinic	triclinic
Space group	$P2_1/c$	P-1
a/Å	18.791(7)	14.8598(8)
b/Å	29.943(18)	18.7258(10)
c/Å	39.805(10)	22.1730(12)
α/°	90	84.875(2)
β/°	91.666(13)	82.374(2)
γ/°	90	72.406(2)
Volume/Å^3	22387(16)	5821.4(5)
Z	8	2
ρ_{calc}g/cm^3	1.264	1.248
μ/mm^{-1}	0.765	0.756
F(000)	8864.0	2272.0
Crystal size/mm^3	0.16 × 0.16 × 0.02	0.16 × 0.12 × 0.08
Radiation	GaKα (λ = 1.34139)	GaKα (λ = 1.34139)
2Θ range for data collection/°	4.64 to 102.618	5.414 to 110.134
Index ranges	$-21 \le h \le 21$, $-34 \le k \le 34$, $-46 \le l \le 36$	$-18 \le h \le 18$, $-21 \le k \le 22$, $-27 \le l \le 26$
Reflections collected	219910	88349
Independent reflections	36776 [R_{int}= 0.0797, R_{sigma}= 0.0532]	22130 [R_{int}= 0.0486, R_{sigma}= 0.0394]
Data/restraints/parameters	36776/0/2777	22130/0/1435
Goodness-of-fit on F^2	1.026	1.052
Final R indexes [I>=2σ (I)]	R_1= 0.0652, wR_2= 0.1827	R_1= 0.0629, wR_2= 0.1695
Final R indexes [all data]	R_1= 0.0935, wR_2= 0.2041	R_1= 0.0838, wR_2= 0.1846
Largest diff. peak/hole / e Å^{-3}	1.17/-1.11	1.45/-1.42

Supplementary Table 5. Crystal data and structure refinement for CB[8]·**G3**$_2$ and CB[8]·**Py**·**G1**.

Compound	CB[8]·**G3**$_2$	CB[8]·**Py**·**G1**
CCDC	2201854	2201856
Empirical formula	$C_{132}H_{126}Cl_4N_{54}O_{24}$	$C_{90}H_{83}Cl_2N_{35}O_{17}$
Formula weight	2994.66	1997.81
Temperature/K	150.00	150.00
Crystal system	monoclinic	triclinic
Space group	P2/n	P-1
a/Å	17.9973(13)	19.571(3)
b/Å	21.3293(15)	25.065(4)
c/Å	46.452(3)	27.220(4)
α/°	90	82.173(7)
β/°	95.958(2)	70.820(7)
γ/°	90	68.868(7)
Volume/Å^3	17735(2)	11761(3)
Z	4	4
ρ_{calc}g/cm^3	1.122	1.128
μ/mm^{-1}	0.786	0.702
F(000)	6224.0	4152.0
Crystal size/mm^3	0.16 × 0.12 × 0.02	0.16 × 0.12 × 0.06
Radiation	GaKα (λ = 1.34139)	GaKα (λ = 1.34139)
2Θ range for data collection/°	4.442 to 96.476	4.4 to 114.4
Index ranges	-18 ≤ h ≤ 19, -19 ≤ k ≤ 23, -51 ≤ l ≤ 51	-24 ≤ h ≤ 23, -31 ≤ k ≤ 31, -34 ≤ l ≤ 34
Reflections collected	109091	153988
Independent reflections	24936 [R_{int}= 0.1034, R_{sigma}= 0.1042]	47933 [R_{int}= 0.0723, R_{sigma}= 0.0653]
Data/restraints/parameters	24936/1/1931	47933/628/2796
Goodness-of-fit on F^2	1.055	1.041
Final R indexes [I>=2σ (I)]	R_1= 0.0843, wR_2= 0.2292	R_1 = 0.0742, wR_2 = 0.2225
Final R indexes [all data]	R_1= 0.1267, wR_2= 0.2580	R_1= 0.1099, wR_2= 0.2457
Largest diff. peak/hole / e Å^{-3}	0.43/-0.74	1.12/-1.02

Theoretical calculations

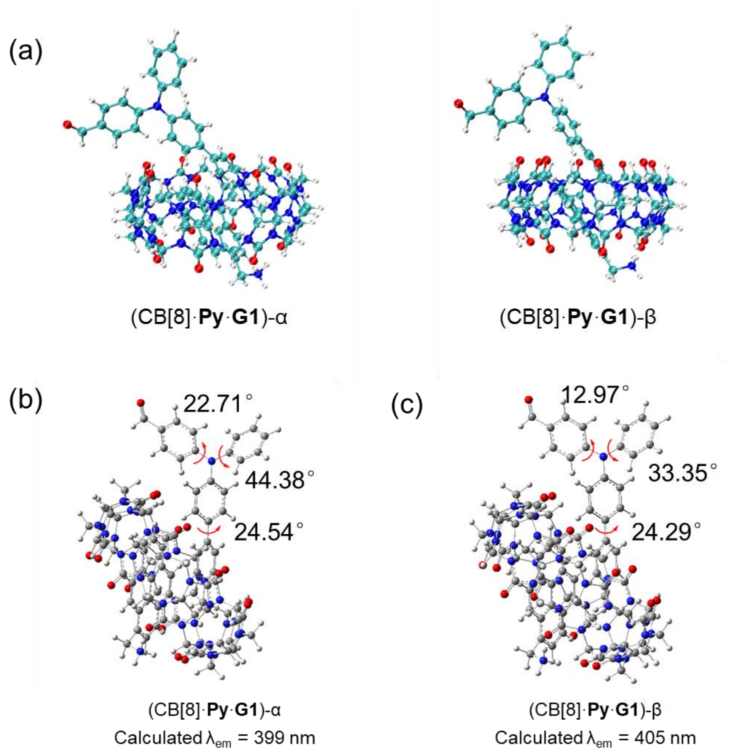

Supplementary Figure 51. (a) Optimized structures of CB[8]·**Py**·**G1** (CB[8]·**Py**·**G1**-α and CB[8]·**Py**·**G1**-β). (b) Conformation of **G1** in CB[8]·**Py**·**G1**-α (Calculated λ_{em} = 399 nm). (c) Conformation of **G1** in (CB[8]·**Py**·**G1**)-β (Calculated λ_{em} = 405 nm).

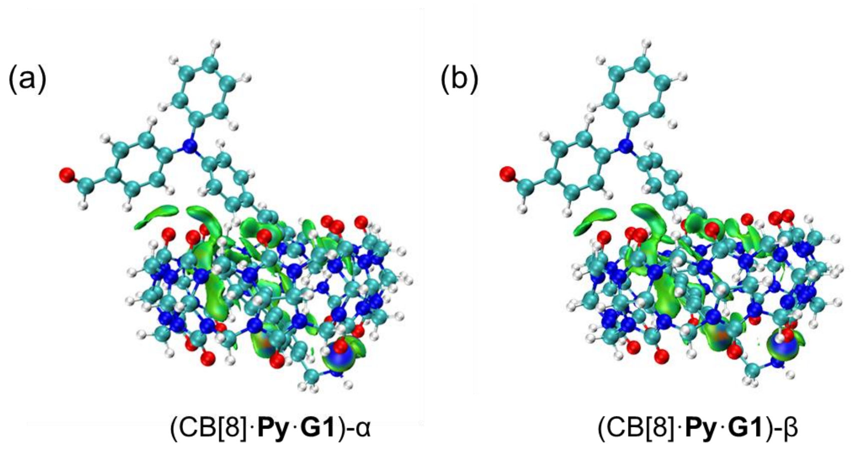

Supplementary Figure 52. Independent gradient model (IGM) analysis for CB[8]·**Py**·**G1**-α (a) and CB[8]·**Py**·**G1**-β (b). The green surfaces represent the noncovalent interactions.

Supplementary Table 6. Cartesian coordinates of optimized (CB[8]·**Py**·**G1**)-α

O	23.9681	24.6126	48.0199
O	33.009	26.4601	47.2394
O	33.3856	28.0033	44.0324
O	26.6159	23.8324	50.2938
O	25.8713	29.4388	52.6207
O	30.3585	24.576	49.8601
O	30.4881	28.7221	41.7215
O	29.9104	30.1003	52.588
O	32.9314	31.7095	50.4336
O	22.4174	31.6626	47.1229
N	25.132	25.1335	51.4927
O	22.6853	30.0254	50.5351
N	27.2992	25.2226	51.9956
O	26.6801	27.9136	42.2717
N	34.3715	29.4424	45.5403
O	32.6108	33.2808	47.1237
N	24.8322	27.378	52.3868
O	24.0036	26.191	44.7404
O	25.3992	32.9765	45.2253
N	27.0014	27.4423	52.9589
O	29.5283	33.6778	45.1837
N	33.3229	30.3009	43.7832
N	31.3882	30.7453	42.364
N	23.2689	25.5489	50.004
N	22.8893	27.7564	50.9502
N	29.6753	25.6002	51.812
N	34.2284	28.3594	47.6972
N	31.5166	26.346	50.8105
N	29.3941	27.8403	52.7155
N	29.2349	30.677	41.8012
N	33.1886	27.3445	49.378
N	21.8089	29.4423	46.9128
N	22.3707	26.2795	48.1066
N	22.599	30.5005	45.1402
N	22.3441	27.2804	45.9146
N	33.0049	32.376	45.0298
N	34.1541	30.4439	48.9379
N	24.3598	31.4993	43.7753
N	33.1756	29.4214	50.6403
N	21.8357	28.4238	49.113
N	31.4211	28.4639	52.0001
N	26.8534	30.1949	41.9312

N	31.0103	32.7634	43.686
N	24.9482	29.3768	42.7359
N	23.3229	28.3529	44.2281
N	34.0762	31.5272	46.7806
N	26.45	32.131	43.3369
N	28.8453	32.584	43.2566
C	26.3637	24.6406	51.1737
C	25.9059	28.2132	52.6457
C	33.6596	29.1264	44.4101
C	26.6747	26.0287	53.0249
H	26.90115	25.63272	54.03118
C	25.1581	25.9861	52.6583
H	24.50962	25.589	53.45942
C	30.5008	25.4084	50.735
C	28.6216	24.6761	52.1592
H	28.70201	23.8181	51.47541
H	28.76157	24.3409	53.2028
C	33.4326	27.2941	48.027
C	23.2658	25.3983	48.6474
C	22.3057	26.5351	50.4476
H	21.6077	26.09546	51.1832
C	23.9164	24.6098	50.9002
H	23.21137	24.32325	51.69819
H	24.20592	23.73089	50.30702
C	22.2815	30.6429	46.4531
C	28.209	27.9835	53.5553
H	28.38605	27.47155	54.51875
H	28.05198	29.04958	53.74412
C	23.4656	27.8709	52.288
H	23.47481	28.94204	52.53231
H	22.83871	27.31847	53.00906
C	30.1618	26.604	52.719
H	30.24146	26.20538	53.74684
C	34.9124	28.4236	46.4181
H	35.9846	28.61445	46.59431
H	34.77297	27.45984	45.91029
C	32.7761	30.3233	42.4331
H	33.39239	30.99556	41.81086
H	32.82298	29.29667	42.04338
C	33.3807	30.6288	50.047
C	30.3764	29.9224	41.94
C	34.6748	30.8488	45.6399
H	35.76719	31.0171	45.6233
C	21.586	28.5084	45.8224

H	20.50771	28.29217	45.70923
C	30.212	28.9271	52.4583
C	33.9286	31.4477	44.4226
H	34.59104	31.97402	43.71041
C	34.6713	29.0995	48.8613
H	35.77455	29.09453	48.92808
C	31.5156	27.0265	52.0965
H	32.39649	26.73787	52.69797
C	22.2159	29.2212	44.6103
H	21.525	29.34144	43.75517
C	21.6199	26.9994	49.1236
H	20.54386	26.75657	49.06271
C	22.4915	28.8504	50.2366
C	33.1665	32.4685	46.3788
C	32.7489	26.1436	50.0825
H	32.56377	25.3649	49.32991
H	33.54751	25.81685	50.77565
C	29.4777	32.0762	42.0457
H	29.1845	32.68501	41.17059
C	21.9225	26.1665	46.7297
H	22.37947	25.25828	46.31215
H	20.82204	26.09094	46.70778
C	25.4043	32.2825	44.2186
C	23.2919	27.1811	44.9338
C	26.2134	29.05	42.3084
C	33.9612	28.3743	50.0324
H	34.65683	27.92991	50.76865
C	30.988	32.1374	42.3772
H	31.5883	32.72235	41.6563
C	32.2252	33.3172	44.262
H	32.84762	33.72547	43.44749
H	31.92217	34.1161	44.954
C	28.0491	30.2164	41.1096
H	28.24752	29.18467	40.78674
H	27.86192	30.86242	40.23383
C	23.9009	28.3823	42.9041
H	23.10071	28.56904	42.16297
H	24.36146	27.40138	42.72148
C	34.6507	31.5266	48.1145
H	35.74938	31.46113	48.03955
H	34.35058	32.46429	48.6038
C	29.763	33.0632	44.1471
C	21.1447	29.3226	48.1981
H	20.11134	28.96221	48.05453

H	21.1434	30.32224	48.65463
C	24.6578	30.7971	42.5603
H	23.86554	30.94706	41.80472
C	26.0464	31.3784	42.1629
H	26.02693	32.01939	41.26302
C	23.0152	31.6291	44.3058
H	22.30643	31.73704	43.46713
H	22.99138	32.51592	44.95355
C	32.6045	29.2939	51.9602
H	32.32603	30.30667	52.28262
H	33.35471	28.87145	52.65374
C	27.4923	33.1359	43.2342
H	27.39989	33.7951	44.10888
H	27.35323	33.71434	42.30077
C	28.4152	30.22002	47.2451
C	27.50811	30.74387	48.21486
C	28.84688	29.09505	45.11956
H	28.49087	28.58444	44.22185
C	27.93248	29.56087	46.07124
C	29.81713	30.37124	47.43855
C	25.667	29.899	46.8365
H	24.59529	29.76187	46.70581
C	30.2763	31.0375	48.6087
H	31.34131	31.22502	48.7257
C	27.99231	31.41305	49.38465
C	26.52194	29.38985	45.91204
H	26.14609	28.85056	45.04312
C	29.41612	31.52896	49.5482
H	29.84009	32.04313	50.41165
C	26.10658	30.60839	47.99742
C	30.70648	29.88217	46.47294
H	31.78062	29.98926	46.6296
C	27.04077	31.94465	50.29173
C	30.2102	29.2681	45.3332
H	30.90334	28.87721	44.58671
C	25.66827	31.80028	50.04838
H	24.94422	32.21263	50.75525
C	25.19631	31.14763	48.91799
H	24.12441	31.08201	48.738
C	27.4527	32.64105	51.55321
H	28.3919	33.20089	51.46257
H	26.66721	33.33207	51.88498
N	27.64248	31.63406	52.66698
H	28.52834	31.08517	52.55389

H	26.87379	30.9264	52.66685
H	27.68056	32.0836	53.58846
N	32.1825	23.9531	41.443
O	35.76256	27.73586	37.59725
N	27.3775	26.6432	47.8967
C	29.7593	25.29133	44.65522
C	31.37881	24.38378	42.52355
C	32.81218	24.90861	40.63724
C	28.93423	25.74056	45.76734
C	29.2777	24.3474	43.7321
H	28.27003	23.9418	43.83512
C	32.2222	22.023	39.9424
H	32.02766	22.68458	39.0965
C	32.32029	22.55859	41.22499
C	31.86202	25.34115	43.43111
H	32.85702	25.765	43.30924
C	33.9556	24.5885	39.8817
H	34.36953	23.58136	39.92498
C	31.07	25.7737	44.4809
H	31.49929	26.50181	45.16696
C	30.07677	23.87868	42.6971
H	29.69491	23.13041	42.00132
C	32.36348	20.65063	39.733
H	32.28373	20.251	38.71968
C	32.3012	26.2168	40.5744
H	31.4075	26.50071	41.12682
C	34.58117	25.55493	39.10684
H	35.47931	25.31646	38.5332
C	29.5107	26.2541	46.9465
H	30.59376	26.29217	47.06602
C	34.08158	26.86823	39.05346
C	27.5259	25.6885	45.7369
H	26.98751	25.33652	44.85754
C	32.58944	19.79201	40.81233
H	32.69584	18.71748	40.65034
C	32.5523	21.6998	42.3035
H	32.63887	22.11593	43.30909
C	28.72667	26.71162	47.97241
H	29.14327	27.14132	48.88184
C	32.93147	27.17696	39.79467
H	32.50386	28.18267	39.75202
C	26.77406	26.12698	46.79888
H	25.68546	26.06641	46.79858
C	34.75381	27.90129	38.24544

H	34.24812	28.90536	38.27608
C	32.67749	20.3254	42.10092
H	32.85932	19.67016	42.95575
C	26.55026	27.14264	48.99985
H	26.16344	28.14125	48.7558
H	25.72069	26.44902	49.16477
H	27.1605	27.19692	49.90823

Supplementary Table 7 Cartesian coordinates of optimized (CB[8]·**Py**·**G1**)-β

O	23.9681	24.6126	48.0199
O	33.009	26.4601	47.2394
O	33.3856	28.0033	44.0324
O	26.6159	23.8324	50.2938
O	25.8713	29.4388	52.6207
O	30.3585	24.576	49.8601
O	30.4881	28.7221	41.7215
O	29.9104	30.1003	52.588
O	32.9314	31.7095	50.4336
O	22.4174	31.6626	47.1229
N	25.132	25.1335	51.4927
O	22.6853	30.0254	50.5351
N	27.2992	25.2226	51.9956
O	26.6801	27.9136	42.2717
N	34.3715	29.4424	45.5403
O	32.6108	33.2808	47.1237
N	24.8322	27.378	52.3868
O	24.0036	26.191	44.7404
O	25.3992	32.9765	45.2253
N	27.0014	27.4423	52.9589
O	29.5283	33.6778	45.1837
N	33.3229	30.3009	43.7832
N	31.3882	30.7453	42.364
N	23.2689	25.5489	50.004
N	22.8893	27.7564	50.9502
N	29.6753	25.6002	51.812
N	34.2284	28.3594	47.6972
N	31.5166	26.346	50.8105
N	29.3941	27.8403	52.7155
N	29.2349	30.677	41.8012
N	33.1886	27.3445	49.378
N	21.8089	29.4423	46.9128
N	22.3707	26.2795	48.1066

N	22.599	30.5005	45.1402
N	22.3441	27.2804	45.9146
N	33.0049	32.376	45.0298
N	34.1541	30.4439	48.9379
N	24.3598	31.4993	43.7753
N	33.1756	29.4214	50.6403
N	21.8357	28.4238	49.113
N	31.4211	28.4639	52.0001
N	26.8534	30.1949	41.9312
N	31.0103	32.7634	43.686
N	24.9482	29.3768	42.7359
N	23.3229	28.3529	44.2281
N	34.0762	31.5272	46.7806
N	26.45	32.131	43.3369
N	28.8453	32.584	43.2566
C	26.3637	24.6406	51.1737
C	25.9059	28.2132	52.6457
C	33.6596	29.1264	44.4101
C	26.6747	26.0287	53.0249
H	26.89925	25.63138	54.03275
C	25.1581	25.9861	52.6583
H	24.50939	25.58852	53.46078
C	30.5008	25.4084	50.735
C	28.6216	24.6761	52.1592
H	28.70211	23.81854	51.47195
H	28.76238	24.3386	53.2039
C	33.4326	27.2941	48.027
C	23.2658	25.3983	48.6474
C	22.3057	26.5351	50.4476
H	21.60665	26.09644	51.18454
C	23.9164	24.6098	50.9002
H	23.21056	24.32007	51.699
H	24.20652	23.73009	50.30519
C	22.2815	30.6429	46.4531
C	28.209	27.9835	53.5553
H	28.38578	27.47263	54.52115
H	28.05056	29.05204	53.74257
C	23.4656	27.8709	52.288
H	23.47482	28.94317	52.53532
H	22.83753	27.31732	53.00987
C	30.1618	26.604	52.719
H	30.24393	26.20557	53.74829
C	34.9124	28.4236	46.4181
H	35.98706	28.61265	46.59209

H	34.76988	27.45771	45.91126
C	32.7761	30.3233	42.4331
H	33.39541	30.99033	41.80519
H	32.81917	29.29039	42.05679
C	33.3807	30.6288	50.047
C	30.3764	29.9224	41.94
C	34.6748	30.8488	45.6399
H	35.76818	31.01733	45.62257
C	21.586	28.5084	45.8224
H	20.50653	28.2926	45.70889
C	30.212	28.9271	52.4583
C	33.9286	31.4477	44.4226
H	34.59368	31.97316	43.71047
C	34.6713	29.0995	48.8613
H	35.77561	29.09473	48.92943
C	31.5156	27.0265	52.0965
H	32.39661	26.73715	52.70028
C	22.2159	29.2212	44.6103
H	21.52195	29.34134	43.7559
C	21.6199	26.9994	49.1236
H	20.54251	26.7563	49.0653
C	22.4915	28.8504	50.2366
C	33.1665	32.4685	46.3788
C	32.7489	26.1436	50.0825
H	32.56084	25.36517	49.32731
H	33.54997	25.81331	50.77407
C	29.4777	32.0762	42.0457
H	29.1854	32.68449	41.16837
C	21.9225	26.1665	46.7297
H	22.38053	25.25678	46.31191
H	20.82024	26.09033	46.70706
C	25.4043	32.2825	44.2186
C	23.2919	27.1811	44.9338
C	26.2134	29.05	42.3084
C	33.9612	28.3743	50.0324
H	34.65856	27.93133	50.7697
C	30.988	32.1374	42.3772
H	31.5878	32.72268	41.65448
C	32.2252	33.3172	44.262
H	32.84903	33.73042	43.44829
H	31.92055	34.11731	44.95473
C	28.0491	30.2164	41.1096
H	28.24644	29.18229	40.78743
H	27.86314	30.86114	40.23043

C	23.9009	28.3823	42.9041
H	23.10122	28.56477	42.15919
H	24.36405	27.39939	42.72549
C	34.6507	31.5266	48.1145
H	35.75136	31.46231	48.0401
H	34.3524	32.46645	48.60476
C	29.763	33.0632	44.1471
C	21.1447	29.3226	48.1981
H	20.10981	28.96131	48.05401
H	21.14155	30.32384	48.65551
C	24.6578	30.7971	42.5603
H	23.86524	30.94641	41.80328
C	26.0464	31.3784	42.1629
H	26.0265	32.02056	41.26219
C	23.0152	31.6291	44.3058
H	22.30472	31.74031	43.46659
H	22.99233	32.51725	44.95501
C	32.6045	29.2939	51.9602
H	32.3243	30.30812	52.28305
H	33.35666	28.87315	52.65512
C	27.4923	33.1359	43.2342
H	27.40058	33.79334	44.1129
H	27.35268	33.71882	42.30171
C	28.41807	30.23875	47.23286
C	27.5085	30.7646	48.20659
C	28.84576	29.10899	45.11991
H	28.48607	28.58476	44.22773
C	27.93618	29.58192	46.06705
C	29.81295	30.37808	47.42912
C	25.667	29.899	46.8365
H	24.59223	29.74155	46.72271
C	30.2763	31.0375	48.6087
H	31.34699	31.20974	48.72953
C	27.99166	31.42723	49.37154
C	26.51896	29.39813	45.91508
H	26.14732	28.83525	45.05433
C	29.42269	31.53523	49.53924
H	29.8502	32.04919	50.40456
C	26.11326	30.62464	47.99309
C	30.70166	29.88234	46.47233
H	31.7793	29.97781	46.63924
C	27.04557	31.95722	50.27768
C	30.2102	29.2681	45.3332
H	30.90368	28.86002	44.59055

C	25.67602	31.8152	50.03699
H	24.94991	32.22915	50.74422
C	25.20544	31.15991	48.91028
H	24.13001	31.0873	48.73421
C	27.45323	32.65102	51.54497
H	28.38951	33.22067	51.45989
H	26.66291	33.33829	51.87964
N	27.64446	31.6458	52.64833
H	28.53108	31.09892	52.52787
H	26.87532	30.93599	52.65249
H	27.68819	32.09544	53.57052
N	32.1825	23.9531	41.443
O	35.56901	27.57821	37.3347
N	27.3775	26.6432	47.8967
C	29.75048	25.29834	44.66486
C	31.39027	24.37319	42.53802
C	32.80546	24.90083	40.63651
C	28.9424	25.73696	45.75714
C	29.2777	24.3474	43.7321
H	28.26794	23.9415	43.83215
C	32.42926	22.04635	39.93235
H	32.31062	22.69849	39.06518
C	32.3221	22.56803	41.22398
C	31.87853	25.32343	43.4662
H	32.89546	25.71332	43.38487
C	33.82292	24.54136	39.72595
H	34.21564	23.52575	39.70572
C	31.07	25.7737	44.4809
H	31.49608	26.50335	45.17264
C	30.06536	23.88692	42.6987
H	29.66638	23.16064	41.98463
C	32.58315	20.68165	39.72649
H	32.64417	20.29844	38.70429
C	32.44031	26.25894	40.7292
H	31.63357	26.59175	41.38435
C	34.42735	25.48806	38.92528
H	35.23204	25.20596	38.2407
C	29.5107	26.2541	46.9465
H	30.59513	26.28487	47.07782
C	34.03349	26.83429	38.99081
C	27.5259	25.6885	45.7369
H	26.97867	25.33956	44.85848
C	32.63124	19.80448	40.81222
H	32.75294	18.73022	40.64958

C	32.3428	21.67704	42.30813
H	32.26172	22.07274	43.32383
C	28.73752	26.70587	47.96713
H	29.15729	27.12615	48.88314
C	33.03924	27.19901	39.90087
H	32.69377	28.23642	39.9563
C	26.77884	26.12651	46.78556
H	25.68791	26.07484	46.78097
C	34.68801	27.83831	38.11119
H	34.30266	28.88493	38.21928
C	32.5012	20.31272	42.10479
H	32.5284	19.64239	42.96798
C	26.56224	27.13315	48.99256
H	26.19268	28.15437	48.79544
H	25.70643	26.45936	49.14061
H	27.1653	27.14365	49.91312

Supplementary References

[1] A. J. Sindt, B. A. DeHaven, D. W. Goodlett, J. O. Hartel, P. J. Ayare, Y. Du, M. D. Smith, A. K. Mehta, A. M. Brugh, M. D. E. Forbes, C. R. Bowers, A. K. Vannucci, L. S. Shimizu, *J. Am. Chem. Soc.* **2020**, *142*, 502-511.

[2] Z. Yang, W. Yin, S. Zhang, I. Shah, B. Zhang, S. Zhang, Z. Li, Z. Lei, H. Ma, *ACS Appl. Bio Mater.* **2020**, *3*, 1187-1196.

[3] P. Yin, T. Wang, Y. Yang, W. Yin, S. Zhang, Z. Yang, C. Qi, H. Ma, *New J. Chem.* **2019**, *43*, 18251-18258.

[4] A. Day, A. P. Arnold, R. J. Blanch, B. Snushall, *J. Org. Chem.* **2001**, *66*, 8094-8100.
